# Cancer-associated fibroblasts stimulate primary tumor growth and metastatic spread in an orthotopic prostate cancer xenograft model

**DOI:** 10.1038/s41598-020-69424-x

**Published:** 2020-07-28

**Authors:** Johannes Linxweiler, Turkan Hajili, Christina Körbel, Carolina Berchem, Philip Zeuschner, Andreas Müller, Michael Stöckle, Michael D. Menger, Kerstin Junker, Matthias Saar

**Affiliations:** 1 Department of Urology and Pediatric Urology, Saarland University, Kirrberger Straße 100, Gebäude 6, 66424 Homburg/Saar, Germany; 2 Institute for Clinical and Experimental Surgery, Saarland University, Homburg/Saar, Germany; 3 Department of Diagnostic and Interventional Radiology, Saarland University, Homburg/Saar, Germany

**Keywords:** Cancer, Medical research, Pathogenesis, Urology

## Abstract

The unique microenvironment of the prostate plays a crucial role in the development and progression of prostate cancer (PCa). We examined the effects of cancer-associated fibroblasts (CAFs) on PCa progression using patient-derived fibroblast primary cultures in a representative orthotopic xenograft model. Primary cultures of CAFs, non-cancer-associated fibroblasts (NCAFs) and benign prostate hyperplasia-associated fibroblasts (BPHFs) were generated from patient-derived tissue specimens. These fibroblasts were coinjected together with cancer cells (LuCaP136 spheroids or LNCaP cells) in orthotopic PCa xenografts to investigate their effects on local and systemic tumor progression. Primary tumor growth as well as metastatic spread to lymph nodes and lungs were significantly stimulated by CAF coinjection in LuCaP136 xenografts. When NCAFs or BPHFs were coinjected, tumor progression was similar to injection of tumor cells alone. In LNCaP xenografts, all three fibroblast types significantly stimulated primary tumor progression compared to injection of LNCaP cells alone. CAF coinjection further increased the frequency of lymph node and lung metastases. This is the first study using an orthotopic spheroid culture xenograft model to demonstrate a stimulatory effect of patient-derived CAFs on PCa progression. The established experimental setup will provide a valuable tool to further unravel the interacting mechanisms between PCa cells and their microenvironment.

## Introduction

Prostate cancer (PCa) is the most frequently diagnosed malignant tumor and the second-leading cause of cancer-related death in men in developed countries^1–3^. Patients diagnosed at an early, organ-confined stage can mostly be cured by radical prostatectomy or radiotherapy. However, many patients still present with or progress to metastatic disease^4,5^, and palliative androgen deprivation therapy (ADT) in combination with the chemotherapeutic agent docetaxel or the androgen-receptor-signaling inhibitors abiraterone or apalutamide is the standard treatment for this condition^6–10^. Virtually all patients acquire resistance to ADT after one to ten years progressing to castration-resistant prostate cancer (CRPC), for which several life-prolonging palliative treatment options are available^11^. In addition to its reliance on androgen receptor signaling from organ-confined to metastatic, castration-resistant disease^12,13^, other hallmarks of PCa are its multifocality^14^, its multiclonality^15,16^ and its notable inter- and intraindividual heterogeneity^17–20^; due to these qualities, PCa management is a major challenge, and thus, further elucidation of the unique biology of this disease is urgently needed. The unique microenvironment (known as the “tumor microenvironment” or “TME”) of the prostate concerning its cellular constitution and the concentration of cytokines, growth factors and hormones is an important factor in PCa development and progression^21,22^. Regarding the fact that the occurrence of metastases is the most important prognostic factor for PCa patients, it would be highly desirable to better understand the contribution of different TME components on metastatic PCa progression and how they could serve as prognostic markers or therapeutic targets. The PCa TME comprises various components, one of the most important and most intensely studied of which are cancer-associated fibroblasts (CAFs). The contribution of CAFs to PCa development and progression was demonstrated by several elegant early studies. Olumi et al. showed that the normally non-tumorigenic benign prostate hyperplasia (BPH) cell line BPH1 can give rise to PCa-resembling tumors when grafted under the renal capsule of immunodeficient mice together with prostate CAFs^23^. Gleave and colleagues observed stimulation of PCa growth and progression when PCa cells were inoculated together with prostate or bone fibroblasts in subcutaneous xenografts^24^. Since then, the number of publications on CAFs and their role in PCa has grown exponentially. However, the biology of CAF-PCa cell interaction and its implications for the management of PCa are still incompletely understood, which might in part be due to the use of suboptimal in vitro and especially in vivo model systems.

In previous work, we successfully established orthotopic PCa xenografts that realistically display the intraprostatic growth of PCa and the development of metastases^25,26^. In this study, we aimed to combine this orthotopic in-vivo model system with three-dimensional PCa spheroid cultures^27,28^ and different patient-derived prostate fibroblast primary cultures to investigate the effect of the latter on PCa progression and metastasis.

## Methods

### Cell culture

LNCaP cells (purchased from DSMZ, Braunschweig, Germany; identity verified by STR profiling) were cultured at 37 °C in RPMI medium (Thermo Fisher, Waltham, MA, USA) supplemented with 10% fetal bovine serum (Biochrom, Berlin, Germany) in a humidified environment with 5% CO_2_. PC3 cells (purchased from DSMZ, Braunschweig, Germany) were cultured at 37 °C in DMEM medium (Thermo Fisher, Waltham, MA, USA) supplemented with 10% fetal bovine serum (Biochrom, Berlin, Germany) in a humidified environment with 5% CO_2_. LuCaP136 spheroids were cultured at 37 °C in ultra-low attachment plates (Corning Inc., Corning, NY, USA) in an optimized StemPro (Thermo Fisher, Waltham, MA, USA) stem cell medium^27,28^ at 37 °C in a humidified environment with 5% CO_2_. Dissociated single cells were regularly removed by filtration with Falcon 40 μm cell strainers (Corning, Inc., Corning, NY, USA), and spheroid viability was regularly tested using a fluorescence-based live/dead assay (Thermo Fisher, Waltham, MA, USA). LuCaP136 spheroids were provided from the Donna Peehl lab (Stanford University, CA, USA). LuCaP136 and LNCaP PCa cells were used in this study the way they were primarily established and are normally cultivated, i.e. LuCaP136 as three-dimensional spheroids and LNCaP as monolayer cultures. Representative microscopic images of LuCaP136 spheroids and LNCaP cells are shown in Supplementary Fig. 1. To count LuCaP136 cells, the spheroids were digested to single cell clusters using Trypsin/EDTA solution (Sigma-Aldrich, St. Louis, MO, USA) under microscopic control and counted with a Neubauer chamber. Thereafter, the volume containing the cell number to be injected was removed and spheroids were allowed to reassemble for 15 min before they were further processed for the preparation of in-vivo experiments.

For the generation of CAF or non-cancer-associated fibroblast (NCAF) primary cultures, tumor-bearing (CAF) or tumor-free (NCAF) tissue pieces were resected from the same radical prostatectomy specimens by an experienced uropathologist immediately after surgery. Sufficient tumor cell content (CAF) or the absence of tumor cells (NCAF) was verified through analyses of H&E-stained frozen sections. Thereafter, the tissue was cut into small pieces (2–3 mm^3^), which were then placed in cell culture flasks and covered with Dulbecco´s modified Eagle’s medium (DMEM; Sigma Aldrich, St. Louis, MO, USA) supplemented with 10% fetal bovine serum (Biochrom, Berlin, Germany) in a humidified environment with 5% CO_2_ at 37 °C. When the fibroblasts began to grow from the tissue pieces, the latter were removed, and these cells were cultivated as conventional monolayer cultures. For the generation of BPH-associated fibroblast (BPHF) primary cultures, tissue pieces from transvesical prostate adenoma enucleation specimens were used and processed as described above for CAF and NCAF primary cultures. The patient characteristics of the fibroblast donors are given in the supplement. All patients from whom samples were obtained to establish fibroblast primary cultures provided informed consent for the use of biological material and clinical data and these experiments were approved by the Ethical Review Board of Saarland (references 188/05 and 141/14). All methods done involving human participants were performed in accordance with the ethical standards of the institutional committee and with the 1964 Helsinki declaration and its later amendments.

For the preparation of cells/spheroids for intraprostatic injection, they were harvested, counted and suspended in a previously prepared, cooled 1:3 mixture of Matrigel HC (Corning, Inc., Corning, NY, USA) and culture medium at a density of 5 × 10^5^ cells/10 μl when the PCa cells were injected alone or at a density of 1 × 10^6^ cells/10 μl (5 × 10^5^ PCa cells plus 5 × 10^5^ fibroblasts) when the PCa cells were injected together with fibroblasts. In the latter case, the medium of the PCa cells (RPMI for LNCaP, StemPro medium for LuCaP136) was used to prepare the cells (PCa cells and fibroblasts) for injection. This suspension was kept on ice until it was drawn up in a cooled 10 μl Hamilton syringe (Hamilton, Reno, NV, USA) immediately before intraprostatic injection.

### Immunofluorescence staining

For characterization of the fibroblast primary cultures by immunofluorescence staining, the cells were seeded into 4-well chamber slides (Corning Falcon, Corning, NY, USA) at a density of 10,000 cells/well. Twenty-four hours later, the medium was removed, the cells were washed three times with phosphate-buffered saline (PBS; Sigma Aldrich, St. Louis, MO, USA) and fixed by 30 min incubation with 4% paraformaldehyde at 37 °C. Thereafter, the cells were washed in PBS, and nonspecific protein binding sites were blocked by incubation in blocking solution (80 ml Tris/HCl pH 7.2, 20 ml FCS, 3 g BSA) for 30 min at room temperature. This step was followed by incubation with primary antibodies diluted in Antibody Diluent (Dako, Glostrup, Denmark) for 1 h at room temperature (mouse anti-Pan-Cytokeratin/1:100/Dako, Glostrup, Denmark; rabbit anti-Vimentin/1:100/Cell Signaling Technology, Danvers, MA, USA; mouse anti-αSMA/1:100/ Cell Signaling Technology, Danvers, MA, USA), washing in PBS and incubation with secondary antibodies diluted in Antibody Diluent (Dako, Glostrup, Denmark) for 1 h at room temperature (goat-anti-mouse IgG-Alexa594/1:1,000; goat-anti-rabbit IgG-Alexa488/1:500; Jackson ImmunoResearch, Cambridgeshire, UK). Finally, after the samples were washed again with PBS, the chambers were removed, the slides were embedded with Vectashield DAPI HardSet mounting medium (Vector Laboratories, Burlingame, CA, USA) and inspected with a Nikon Eclipse Ci fluorescence microscope and a Nikon digital-sight DS-2MBWc camera unit (Nikon, Minato, Japan).

### Animals

Male immunodeficient SCID-mice (CB17/lcr-*Prkdc*^*scid*^/lcrlcoCrl, 8–10 weeks old; Charles River Laboratories, Sulzfeld, Germany) were kept in isolated ventilated cages under specific pathogen-free conditions in a temperature- and humidity-controlled, 12 h dark/light environment at the animal care facility of the Institute for Clinical and Experimental Surgery at Saarland University. The animals had free access to tap water and standard pellet food. Their health status was monitored daily. All experiments were approved by the local governmental animal care committee (No. 30/2015) and conducted in accordance with the German legislation on the protection of animals and the National Institutes of Health Guide for the Care and Use of Laboratory Animals (NIH Publication #85-23 Rev. 1985)^25,26^.

### Orthotopic tumor cell implantation and follow-up

Intraprostatic tumor cell (+ /− fibroblast) injection was performed as previously described^25^. Briefly, after a lower midline incision and preparation of seminal vesicles, prostate and urinary bladder, 10 μl of a 1:3 Matrigel:medium suspension containing 5 × 10^5^ (PCa cells alone) or 1 × 10^6^ (5 × 10^5^ PCa cells combined with 5 × 10^5^ fibroblasts) cells were injected into the left anterior prostate lobe using a cooled 10 μl Hamilton syringe. For analysis of the orthotopic tumor engraftment and growth as well as the development of metastases, all mice underwent high-resolution ultrasonography (hrUS) examinations at 3, 4, 5, 6, 8 and 10 weeks, in vivo micro CT (μCT) analyses at 6, 8 and 10 weeks and 9.4 T MRI analyses at 6 and 10 weeks after intraprostatic tumor cell injection. The imaging studies (hrUS, μCT, MRI) were performed as previously described^25^.

Mice were sacrificed after 10 weeks by cervical dislocation. During autopsy, the prostate tumor was resected for histological analysis, and the abdominal visceral organs, lungs and lymph nodes were inspected for macroscopically visible metastases. In addition to the primary tumor tissue, the lower extremity bones, lungs, liver and the renal as well as lumbar aortic lymph nodes were subjected to histological evaluation. Concerning regional lymph nodes, we routinely removed the sacral, iliac and lumbar lymph nodes (4–6 lymph nodes per mouse).

### Serum PSA measurements

Blood samples were collected from all mice at 4, 6, 8 and 10 weeks after intraprostatic injection via puncture of the retrobulbar sinus under general anesthesia with glass microcapillaries (Sigma Aldrich, St. Louis, MO, USA). The PSA levels in mouse serum were analyzed in the core laboratory facility of Saarland University Medical Center as previously described^26^.

### Immunohistochemistry

Immunohistochemistry of the primary tumors and metastases resected during autopsy was performed as previously described^25^ using human-specific primary antibodies against cytokeratin (1:200/Dako, Glostrup, Denmark), Ki67 (Dako, Glostrup, Denmark; 1:200), vimentin (1:100/Cell Signaling Technology, Danvers, MA, USA) and Ku70 (1:100/Abcam, Cambridge, UK).

### Statistical analyses

Statistical analyses were performed with Excel for Mac V16.15 (Microsoft, Redmond, USA), SigmaPlot Version 13 (Systat Software Inc., San Jose, USA) and SPSS Statistics 23 (IBM, Armonk, USA). In general, two-tailed statistical tests were performed, and *p* values < 0.05 were considered statistically significant. The serum PSA values are shown in boxplots.

## Results

### Natural tumor progression in orthotopic LuCaP136 and LNCaP xenografts

To observe the natural course of primary tumor growth and the development of metastases in “native” LuCaP136 and LNCaP xenografts, we orthotopically injected 5 × 10^5^ LuCaP136 or LNCaP cells in 8 mice for each cell line and monitored local and systemic tumor progression for 10 weeks by small animal imaging analyses and serum PSA measurements. The primary tumors showed exponential growth, reaching a median volume of approximately 120 mm^3^ for the LuCaP136 tumors and approximately 180 mm^3^ for the LNCaP tumors after 10 weeks (Suppl. Figure 2A). Primary tumor growth was accompanied by an exponential increase in serum PSA values, which ranged from 0.1 to 1.5 ng/ml in LuCaP136 xenografts and 5 to 110 ng/ml in LNCaP xenografts (Suppl. Figure 2B). Notably, the primary tumor volumes determined by high-resolution ultrasonography and serum PSA values showed a good correlation, with r^2^ values of 0.87 for LuCaP136 xenografts and 0.85 for LNCaP xenografts (Suppl. Figure 2C). Histologically, both cell lines gave rise to solid tumors composed of relatively monomorphic cells showing a high number of mitotic figures (Suppl. Figure 3). Concerning metastatic seeding, we observed regional (iliac and lumbal) lymph node metastases after 10 weeks in 5 of 6 mice in the LuCaP136 group and 4 of 7 mice in the LNCaP group (Table 1). No metastases in the lungs or other organs were observed in LuCaP136 xenografts, while lung metastases were present in 1 of 7 animals in the LNCaP group. Two mice in the LuCaP136 group and one mouse in the LNCaP group died prematurely due to non-cancer-associated causes.

**Table 1:** Development of lymph node and lung metastases in orthotopic LuCaP136 and LNCaP xenografts. 10 weeks after intraprostatic injection of 5 × 10^5^ LuCaP136 or LNCaP cells, mice were sacrificed, and their organs were examined for the presence of metastases. The number of animals with lymph node and lung metastases is given in this table (based on histological examination of the removed organs). Two animals in the LuCaP136 and one animal in the LNCaP group (initially n = 8 in both groups) died prematurely due to not cancer-specific causes. LN = lymph node.

	No LN metastases	1 LN metastasis	2 LN metastases	≥ 3 LN metastases	No lung metastases	Lung metastases
LuCaP136 alone (n = 6)	1	3	2	0	6	0
LNCaP alone (n = 7)	3	3	1	0	6	1

### Generation and characterization of fibroblast primary cultures

**Figure 1:**
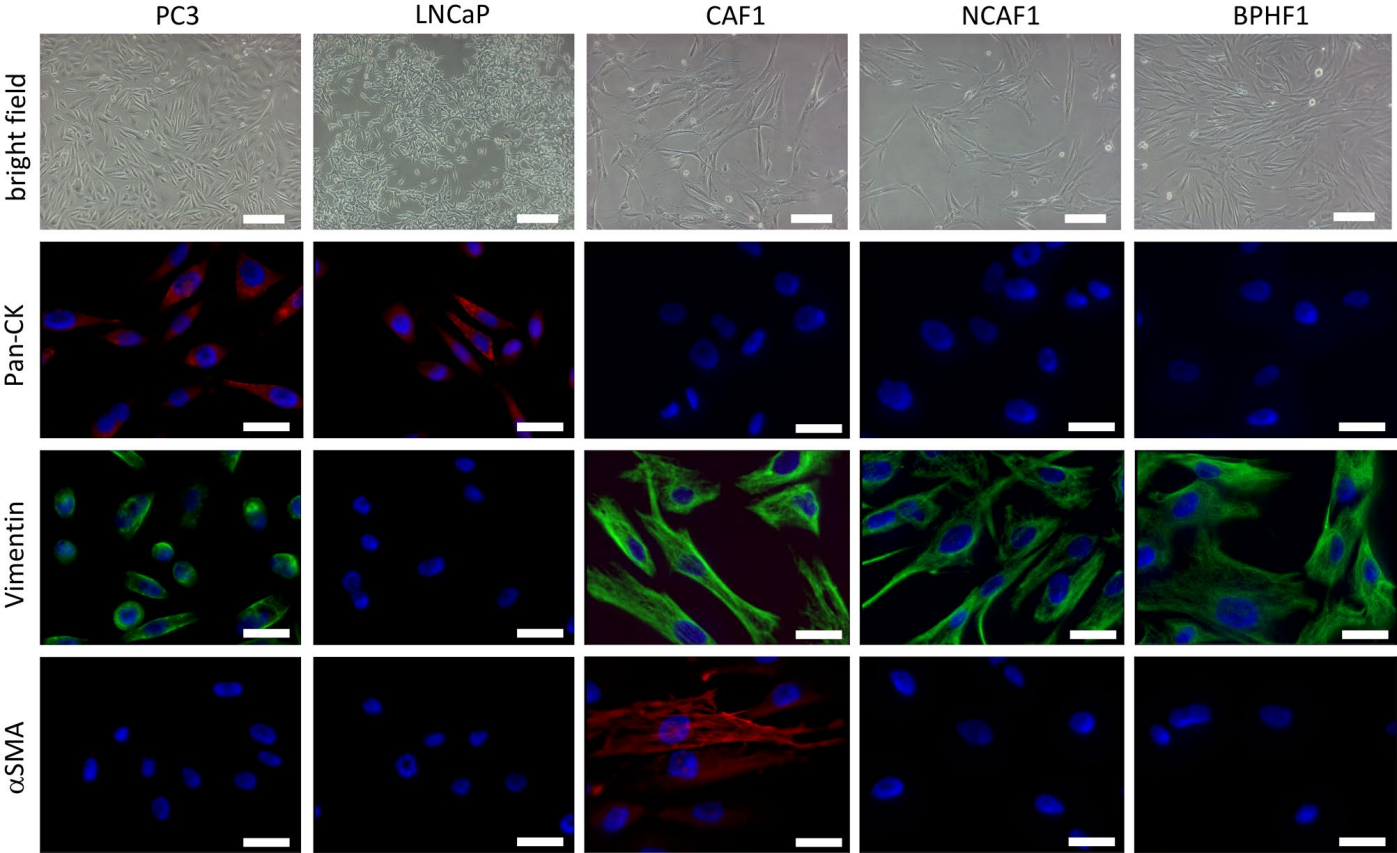
Characterization of the fibroblast primary cultures used for in vivo coinjection series 1 via immunofluorescence staining. The cancer-associated fibroblast (CAF), non-cancer-associated fibroblast (NCAF) and benign prostate hyperplasia-associated fibroblast (BPHF) primary cultures used in the first in vivo coinjection series (CAF1, NCAF1, BPHF1) were characterized by immunofluorescence staining for the markers Pan-cytokeratin, Vimentin and αSMA. PC3 and LNCaP were used as positive controls for vimentin and Pan-CK (PC3 vimentin and Pan-CK double positive, LNCaP Pan-CK positive). Scale bar (bright field pictures) = 50 μm. Scale bar (immunofluorescence pictures) = 20 μm. αSMA = alpha smooth muscle actin, Pan-CK = Pan-Cytokeratin.

CAFs, NCAFs and BPHFs could be successfully generated from cancerous (CAFs) and tumor-free (NCAFs) radical prostatectomy tissue specimens as well as from prostate adenoma enucleation specimens (BPHFs). The success rate to generate fibroblast primary cultures was close to 100%, and these primary cultures could be kept in culture until passage 20 to 30 before they became senescent. However, in the experiments described here, all primary cultures were used in early passages (1 to 7). Before using these cells for in vivo experiments, we characterized the fibroblast primary cultures by immunofluorescence staining. While all fibroblasts stained positive for vimentin and negative for Pan-CK, only CAFs showed αSMA expression to varying degrees (15% to 80% of cells). Figure 1 shows representative images of the fibroblast primary cultures used for the first in vivo coinjection series described in the next section. PC3, which is Pan-CK and vimentin double positive, and LNCaP, which is Pan-CK positive, were used as positive controls. Importantly, in all in vivo experiments, the CAFs and NCAFs used in one in vivo series were always generated from the same radical prostatectomy specimen to avoid interindividual “background noise” in fibroblast biology.

### Effect of orthotopic fibroblast coinjection on tumor progression and the development of metastases

In a next step, we performed orthotopic coinjection of prostate cancer cells (LuCaP136 or LNCaP) with different fibroblast primary cultures (CAFs, NCAFs, BPHFs), resulting in 6 groups with 8 mice each (in vivo coinjection series 1), to examine the effects of fibroblasts on local and systemic PCa progression. We observed stimulatory effects of fibroblast coinjection on tumor progression with CAFs showing the strongest effects in both xenograft models.

**Figure 2:**
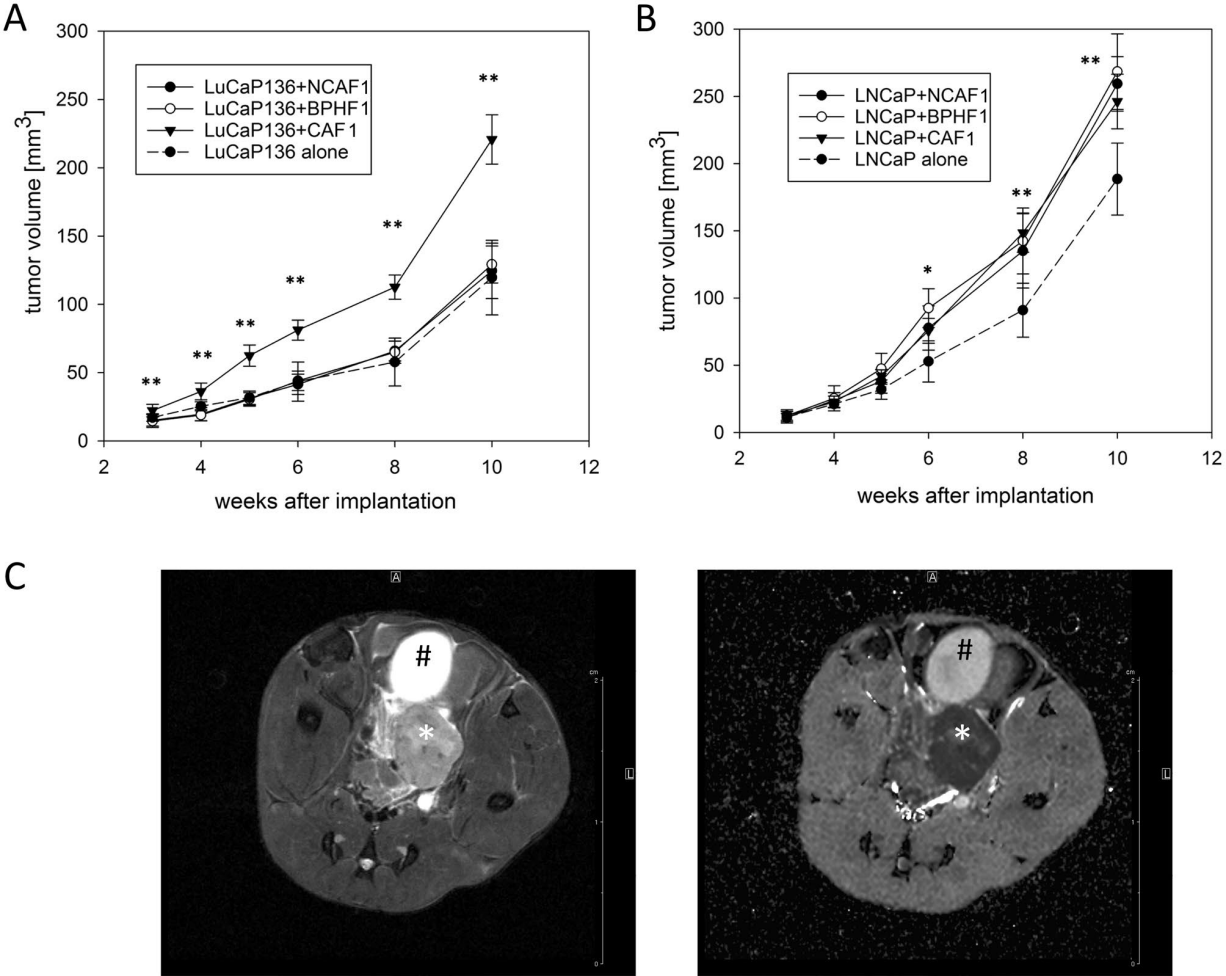
Primary tumor growth after coinjection with different fibroblast primary cultures in orthotopic LuCaP136 and LNCaP xenografts. Primary tumor growth curves after coinjection with cancer-associated fibroblasts (CAF1), non-cancer-associated fibroblasts (NCAF1) or benign prostate hyperplasia-associated fibroblasts (BPHF1) in orthotopic LuCaP136 (**A**) and LNCaP (**B**) xenografts. The dashed lines represent the growth curves when LuCaP136 or LNCaP cells were injected alone. Tumor volumes were determined by high-resolution 3D ultrasonography. *p* values were determined by the Mann–Whitney U test (comparison of each coinjection group vs. tumor cell alone group). *p < 0.05 **p < 0.01 ***p < 0.001. (**C**) Representative transversal T2-weighted (left) and diffusion-weighted (right; ADC-map) MRI-images of orthotopic xenograft tumors (here: LuCaP136 tumor). The urinary bladder is marked with a black rhomb, the tumor with a white asterisk. Scale bar = 2 cm.

In detail, comparing the primary tumor growth curves from this first *in viv*o coinjection series (Fig. 2, continuous lines) with those after injection of LuCaP136 spheroids or LNCaP cells alone (Fig. 2, dashed lines; corresponding to the growth curves in Suppl. Figure 2A), we found that fibroblast coinjection had different effects on primary tumor growth in LuCaP136 versus LNCaP xenografts. In LuCaP136 xenografts, coinjection of CAFs led to significantly higher tumor volumes compared to NCAF and BPHF coinjection. In the latter two cases, primary tumor growth was comparable to that following injection of LuCaP136 spheroids alone (Fig. 2A). In LNCaP xenografts, CAF, NCAF and BPHF coinjection all resulted in significantly increased tumor volumes compared to injection of LNCaP cells alone (Fig. 2B). 9.4 T MRI imaging showed solid intraprostatic tumors with marked diffusion restriction in all cases (Fig. 2C).

**Figure 3:**
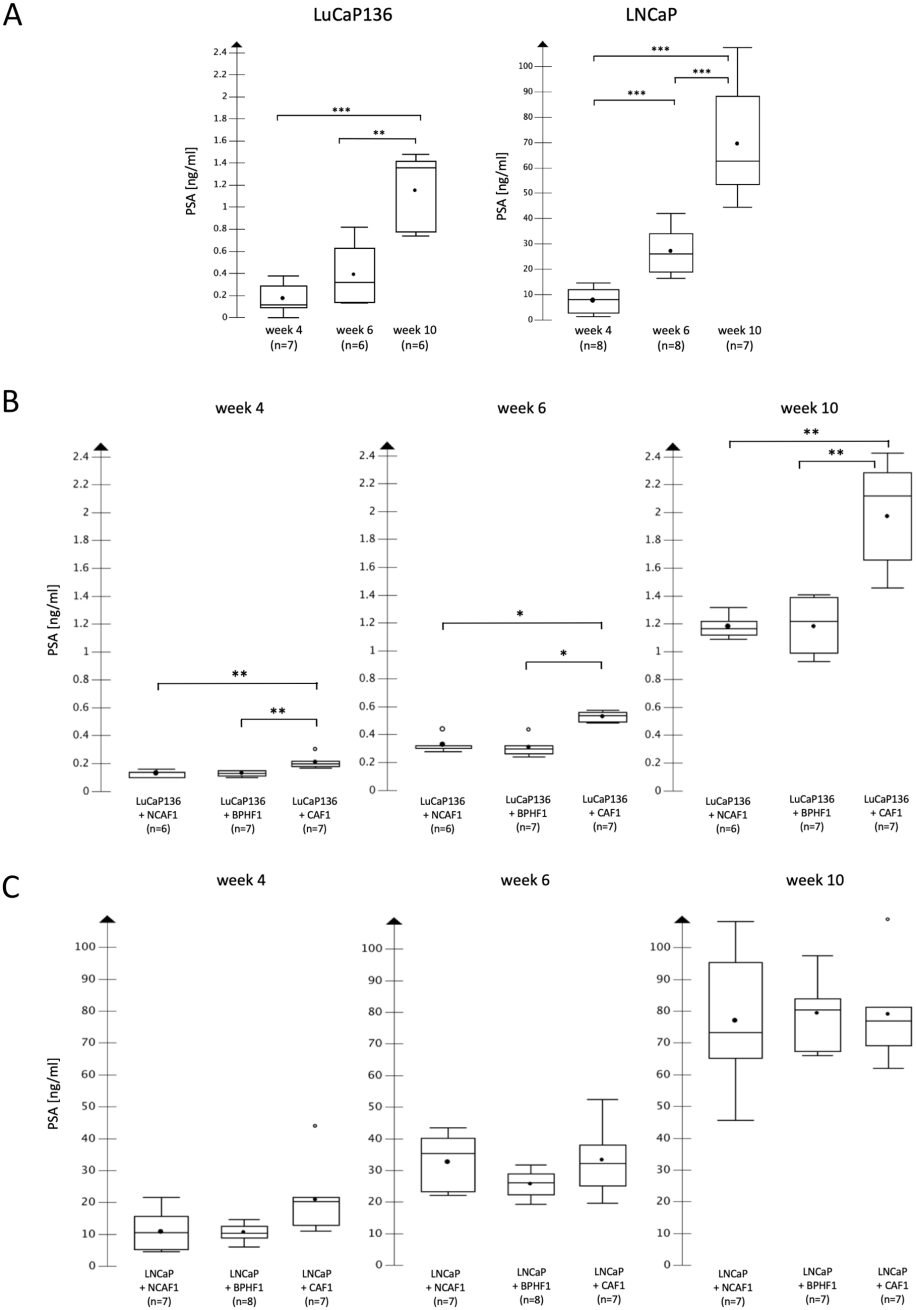
Development of serum PSA values after coinjection with different fibroblast primary cultures in orthotopic LuCaP136 and LNCaP xenografts. Analysis of the serum PSA values after coinjection with cancer-associated fibroblasts (CAF1), non-cancer-associated fibroblasts (NCAF1) or benign prostate hyperplasia-associated fibroblasts (BPHF1) in orthotopic LuCaP136 (**B**) and LNCaP (**C**) xenografts. The results from the LuCaP136 alone and LNCaP alone groups are shown as well for comparison (**A**). *p* values were determined by the Mann–Whitney U test. *p < 0.05 **p < 0.01 ***p < 0.001.

According to the observations in primary tumor volume analyses, serum PSA values were significantly higher in the LuCaP136 + CAF1 group than in the LuCaP136 + NCAF1 group and the LuCaP136 + BPHF1 group (Fig. 3B). The serum PSA values of the latter two groups were comparable to those when LuCaP136 spheroids were injected alone (Fig. 3A). In LNCaP xenografts, again corresponding well to the primary tumor volume results, no difference in serum PSA values could be observed between the different coinjection groups (Fig. 3C). Compared to the injection of LNCaP cells alone (Fig. 3A), serum PSA values after fibroblast coinjection were slightly but not significantly higher.

In addition to the stimulatory effect on primary tumor growth, coinjection of CAFs led to an increased frequency of lymph node and lung metastases in LuCaP136 xenografts compared to both, the injection of LuCaP136 spheroids alone as well as the coinjection of the other two fibroblast types (Table 2). The presence and number of lymph node and lung metastases were confirmed by histopathological examination of the removed organs after autopsy. As with primary tumor volumes and serum PSA values, LNCaP xenografts showed no significant differences in the occurrence of lymph node and lung metastases after coinjection with CAF1, NCAF1 or BPHF1 cells. However, the frequency of lymph node and lung metastases was increased especially in the LNCaP + CAF1 group though these differences were not statistically significant (Table 2). Representative H&E-stained microphotographs of lymph node and lung metastases in LuCaP136 and LNCaP xenografts are shown in supplementary Fig. 4, immunohistochemical stainings proving their human origin as well as representative MRI and CT images in supplementary Fig. 5. Metastases in organs other than the lymph nodes and lungs were not observed in this in vivo series.

**Table 2:** Development of lymph node and lung metastases after coinjection with different fibroblast primary cultures in orthotopic LuCaP136 and LNCaP xenografts. 10 weeks after intraprostatic injection of 5 × 10^5^ LuCaP136 or LNCaP cells combined with 5 × 10^5^ CAF1, NCAF1 or BPHF1 cells, mice were sacrificed, and their organs were examined for the presence of metastases. The number of animals with lymph node and lung metastases in each of the 6 resulting groups is given in this table (based on histological examination of the removed organs). The results from the first series, in which LuCaP136 and LNCaP cells were injected alone (see also Table 1), are added as well. Single animals in different combination groups (initially n = 8 in both groups) died prematurely due to not cancer-specific causes. *p* values were determined by Fisher´s exact test. BPHF = benign prostate hyperplasia associated fibroblasts, CAF = cancer-associated fibroblasts, NCAF = non-cancer-associated fibroblasts, LN = lymph node.

	≥ 2 LN metastases	Statistical significance	Lung metastases	Statistical significance
LuCaP136 alone (n = 6)	2/6	/	0/6	/
LuCaP136 + NCAF1 (n = 6)	1/6	*p* = 1,00 (vs. LuCaP136 alone)	0/6	*p* = 1,00 (vs. LuCaP136 alone)
LuCaP136 + BPHF1 (n = 7)	2/7	*p* = 1,00 (vs. LuCaP136 alone) *p* = 0,63 (vs. LuCaP136 + NCAF1)	1/7	*p* = 1,00 (vs. LuCaP136 alone) *p* = 1,00 (vs. LuCaP136 + NCAF1)
LuCaP136 + CAF1 (n = 7)	6/7	*p* = 0,10 (vs. LuCaP136 alone) *p* **= 0,03 (vs. LuCaP136 + NCAF1)** *p* = 0,10 (vs. LuCaP136 + BPHF1)	4/7	*p* = 0,07 (vs. LuCaP136 alone) *p* = 0,07 (vs. LuCaP136 + NCAF1) *p* = 0,27 (vs. LuCaP136 + BPHF1)
LNCaP alone (n = 7)	1/7	/	1/7	/
LNCaP + NCAF1 (n = 7)	1/7	*p* = 1,00 (vs. LNCaP alone)	2/7	*p* = 1,00 (vs. LNCaP alone)
LNCaP + BPHF1 (n = 7)	2/7	*p* = 1,00 (vs. LNCaP alone) *p* = 1,00 (vs. LNCaP + NCAF1)	2/7	*p* = 1,00 (vs. LNCaP alone) *p* = 1,00 (vs. LNCaP + NCAF1)
LNCaP + CAF1 (n = 7)	4/7	*p* = 0,27 (vs. LNCaP alone) *p* = 0,27 (vs. LNCaP + NCAF1) *p* = 0,59 (vs. LNCaP + BPHF1)	5/7	*p* = 0,10 (vs. LNCaP alone) *p* = 0,29 (vs. LNCaP + NCAF1) *p* = 0,29 (vs. LNCaP + BPHF1)

### Validation of the effects of orthotopic fibroblast coinjection in LuCaP136 xenografts

**Figure 4:**
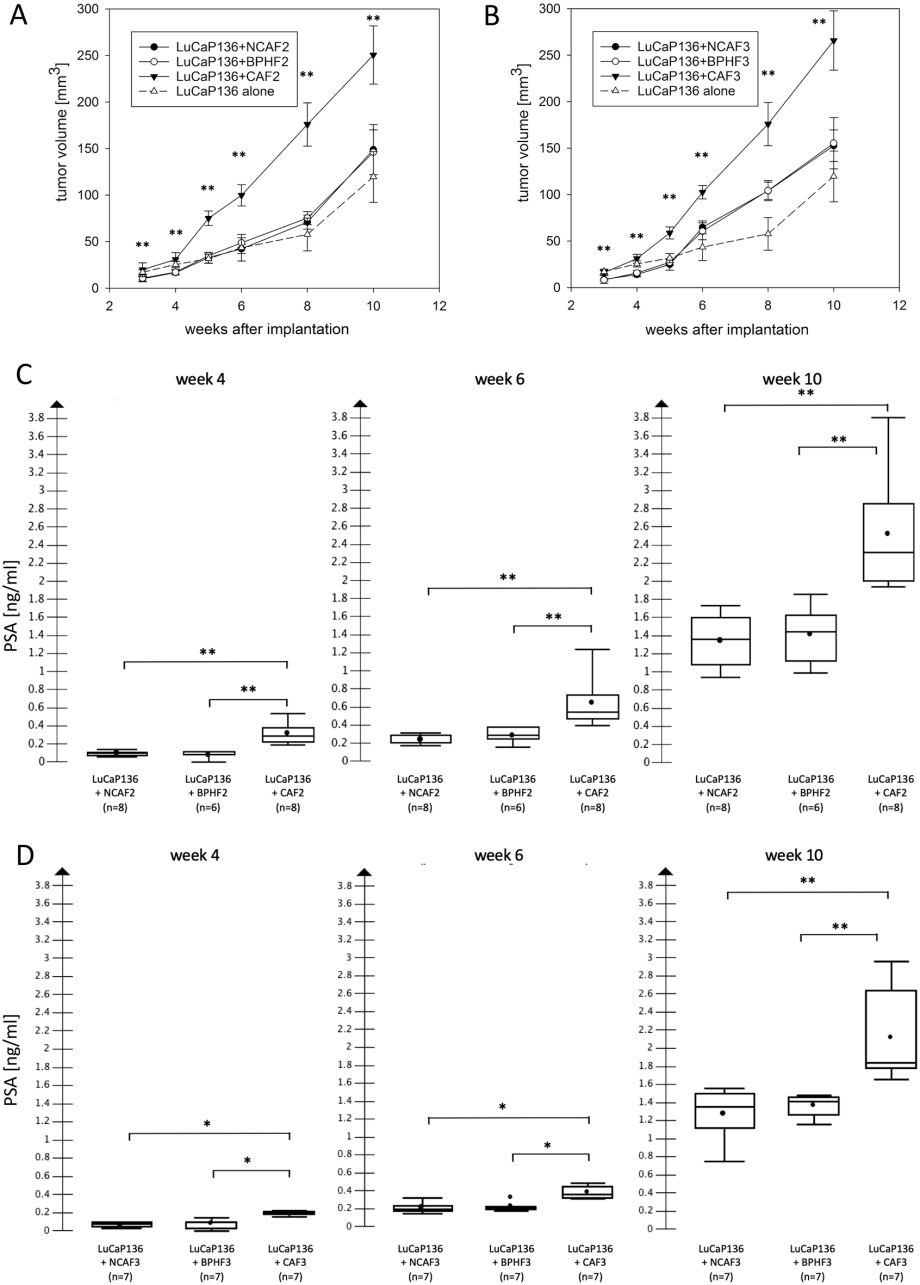
Validation of the effects of orthotopic fibroblast coinjection in LuCaP136 xenografts on primary tumor growth and serum PSA values using two further sets of fibroblast primary cultures [CAF2/NCAF2/BPHF2 (**A** and **C**) and CAF3/NCAF3/BPHF3 (**B** and **D**)]. Primary tumor growth curves (A, B) and serum PSA values (C, D) after coinjection with cancer-associated fibroblasts (CAF), non-cancer-associated fibroblasts (NCAF) or benign prostate hyperplasia-associated fibroblasts (BPHF) in orthotopic LuCaP136 xenografts are shown. The results from the LuCaP136 alone group are shown as well. Tumor volumes were determined via high-resolution 3D ultrasonography. *p* values were determined by the Mann–Whitney U test. *p < 0.05 **p < 0.01 ***p < 0.001.

Since we observed a stimulation of primary tumor growth as well as metastatic spread by CAFs compared to NCAFs and BPHFs in LuCaP136 xenografts in our first in vivo coinjection series, we aimed to validate these results in two further in vivo experiments (in vivo coinjection series 2 and 3) using two other sets of fibroblast primary cultures (CAF2/NCAF2/BPHF2 and CAF3/NCAF3/BPHF3, respectively). All fibroblasts showed negative Pan-CK and positive vimentin staining, while αSMA was only expressed by CAFs (Suppl. Figure 6). In both validation experiments, CAF coinjection again resulted in significantly higher primary tumor volumes and significantly higher serum PSA values compared to NCAF or BPHF coinjection (Fig. 4). In the first validation experiment (in vivo coinjection series 2), the frequency of lymph node metastases was significantly higher in the CAF2 coinjection group compared to the NCAF2/BPHF2/LuCaP136 alone groups (each *p* < 0.05), while in the second validation experiment (in vivo coinjection series 3), metastatic spread to lymph nodes was significantly stimulated in the CAF3 coinjection group compared to the NCAF3 coinjection group (*p* = 0.01; CAF3 vs. BPHF3 and CAF3 vs. LuCaP136 both *p* = 0.10) (Table 3). The frequency of lung metastases was significantly higher in the LuCaP136 + CAF3 group vs. the LuCaP136 + NCAF3 group and the LuCaP136 alone group (both *p* < 0.05) (Table 3).

**Table 3:** Validation of the effect of fibroblast coinjection on the development of lymph node and lung metastases in orthotopic LuCaP136 xenografts: second and third combination in-vivo series. 10 weeks after intraprostatic injection of 5 × 10^5^ LuCaP136 cells combined with 5 × 10^5^ CAF2/CAF3, NCAF2/NCAF3 or BPHF2/BPHF3 cells, mice were sacrificed, and their organs were examined for the presence of metastases. The number of animals with lymph node and lung metastases in each of the 3 resulting groups is given in this table (based on histological examination of the removed organs). The results from the first series, in which LuCaP136 and LNCaP cells were injected alone (see also Table 1), are added as well. *p* values were determined by Fisher´s exact test. BPHF = benign prostate hyperplasia associated fibroblasts, CAF = cancer-associated fibroblasts, NCAF = non-cancer-associated fibroblasts, LN = lymph node.

	≥ 2 LN metastases	Statistical significance	Lung metastases	Statistical significance
LuCaP136 alone (n = 6)	2/6	/	0/6	/
LuCaP136 + NCAF2 (n = 8)	4/8	*p* = 0,63 (vs. LuCaP136 alone)	2/8	*p* = 0,47 (vs. LuCaP136 alone)
LuCaP136 + BPHF2 (n = 6)	3/6	*p* = 1,00 (vs. LuCaP136 alone) *p* = 1,00 (vs. LuCaP136 + NCAF2)	1/6	*p* = 1,00 (vs. LuCaP136 alone) *p* = 1,00 (vs. LuCaP136 + NCAF2)
LuCaP136 + CAF2 (n = 8)	8/8	*p* **= 0,02 (vs. LuCaP136 alone)** *p* **= 0,02 (vs. LuCaP136 + NCAF2)** *p* **= 0,05 (vs. LuCaP136 + BPHF2)**	3/8	*p* = 0,21 (vs. LuCaP136 alone) *p* = 0,32 (vs. LuCaP136 + NCAF2) *p* = 0,58 (vs. LuCaP136 + BPHF2)
LuCaP136 + NCAF3 (n = 7)	1/7	*p* = 0,56 (vs. LuCaP136 alone)	1/7	*p* = 1,00 (vs. LuCaP136 alone)
LuCaP136 + BPHF3 (n = 7)	2/7	*p* = 1,00 (vs. LuCaP136 alone) *p* = 0,51 (vs. LuCaP136 + NCAF3)	3/7	*p* = 0,19 (vs. LuCaP136 alone) *p* = 0,237 (vs. LuCaP136 + NCAF3)
LuCaP136 + CAF3 (n = 7)	6/7	*p* = 0,10 (vs. LuCaP136 alone) *p* **= 0,01 (vs. LuCaP136 + NCAF3)** *p* = 0,10 (vs. LuCaP136 + BPHF3)	5/7	*p* **= 0,02 (vs. LuCaP136 alone)** *p* **= 0,03 (vs. LuCaP136 + NCAF3)** *p* = 0,59 (vs. LuCaP136 + BPHF3)

### Immunohistochemistry of orthotopic tumors after coinjection of fibroblast and PCa cells

Primary tumors harvested during autopsy were subjected to immunohistochemistry using anti-Cytokeratin, anti-Vimentin and human-specific anti-Ku70 antibodies. While the tumor cells proved to be of epithelial and human origin (Cytokeratin positive, Ku70 positive, Vimentin negative), human fibroblasts (expected to be Cytokeratin negative, Ku70 positive, Vimentin positive) could not clearly be detected 10 weeks after injection (n = 5 per xenograft type; Suppl. Figure 7).

## Discussion

For many decades, scientists have mainly focused on cancer cells themselves as the primary target to elucidate the biology of cancer and to develop new preventive, diagnostic or therapeutic approaches. However, the pivotal importance of the so-called tumor microenvironment (TME) including different cellular and extracellular factors, such as blood vessels, lymph vessels, fibroblasts, immune cells and collagen fibers, for nearly all tumor entities, including prostate cancer (PCa), has become increasingly evident in recent years. To date, the most intensely studied PCa TME components are cancer-associated fibroblasts (CAFs). These comprise several fibroblast subtypes that arise through continuous mutual interaction with nearby cancer cells and are characterized by the acquisition of an activated myofibroblast-like phenotype, the expression of specific markers such as αSMA, FAP or TGF-β and other specific molecular changes^29–31^. In contrast, genetic aberrations are generally not observed^32^. In previous work, CAFs have been shown to play an important role in many aspects of PCa biology, such as local tumor growth and progression^24,33,34^, the development of metastases^29,35,36^ and the emergence of drug resistance^37,38^. Accordingly, these cells have also been identified as promising therapeutic targets^39–41^ and as potential prognostic markers^42^. Even so, most of these studies come along with major drawbacks. First, in many studies, immortalized fibroblast cultures or fibroblast cultures that are not truly representative for PCa CAFs were used. Second, in vivo experiments were mostly performed in subcutaneous or renal subcapsular xenografts. In our opinion, when the different biological aspects of prostate cancer-associated fibroblasts are investigated in vivo, it is of utmost importance to use a) fibroblast cultures that are well characterized and of human origin (ideally patient-derived primary cultures) and b) the orthotopic/intraprostatic grafting site, which represents a unique microenvironment with regard to its cellular composition and the local concentration of growth factors and hormones, especially testosterone. We consider the latter point to be crucial since prostate CAFs are known to express an androgen receptor (AR), which plays an important role for many biological effects exerted by these cells^43–46^.

To the best of our knowledge, there is only one recent study in addition to ours that used patient-derived fibroblast primary cultures for coimplantation with PCa cells in an orthotopic xenograft model. Mishra and colleagues prepared cell recombinants by mixing CWR22Rv1 PCa cells with patient-derived CAFs in collagen at a 1:3 ratio^38^. Primary cultures were defined as CAFs when they led to the development of tumors within four weeks after renal subcapsular grafting together with normally non-tumorigenic BPH1 cells (otherwise they were termed NAFs), which is a stringent and straightforward method of CAF characterization dating back to the pioneering work of Olumi, Hayward and colleagues^23,47^. The resulting collagen plugs were then surgically implanted into the anterior prostate lobes, and the tumor volumes were measured by calipers after the end point of the experiment was reached.

In our study, unlike Mishra et al., we generated pairs of fibroblast primary cultures (CAFs and NCAFs) from cancerous and cancer-free tissue areas of the same radical prostatectomy specimens, which were then used for intraprostatic coinjection and compared in our orthotopic xenograft model. Therefore, when the effects of CAFs and NCAFs were compared, the “background” noise in the biology of these two fibroblast populations was minimized compared to that with two entirely different fibroblast cell lines or two cell lines derived from different patients. Another aspect that was different from Mishra et al. and unique in our study is that, besides LNCaP monolayer cells, we also used a three-dimensional spheroid culture as prostate cancer cell component in the orthotopic coinjection experiments. LuCaP136 spheroids were generated from the equally named patient-derived xenografts (PDX) and are characterized by the expression of a wild-type androgen receptor, loss of both *PTEN* alleles, and absence of the *TMPRSS2:ERG* fusion gene. When implanted into the tibiae of immunodeficient mice, LuCaP136 spheroids give rise to osteosclerotic bone metastases, which respond to castration^27,28,48^. Furthermore, we performed repeated noninvasive monitoring of local and systemic tumor burden by small animal imaging analyses and repeated serum PSA measurements, which allows the longitudinal observation of tumor progression instead of end point measurements only.

In our study, looking at the effect of coinjection of different fibroblasts on primary tumor growth and metastatic spread in LuCaP136 and LNCaP xenografts, statistically significant results were observed concerning primary tumor growth in the LuCaP136 + CAF coinjection groups vs. the other three LuCaP136 groups (LuCaP136 alone/LuCaP136 + NCAF/ LuCaP136 + BPHF) in all three in-vivo series in terms of significantly higher primary tumor volumes in the CAF-coinjection groups. In LNCaP xenografts, all coinjection groups (LNCaP + CAF1/ LNCaP + NCAF1/ LNCaP + BPHF1) showed significantly higher primary tumor volumes as compared to the injection of LNCaP alone while there was no difference between the different fibroblast coinjection groups. PSA values were significantly higher in LuCaP136 + CAF vs. LuCaP136 + NCAF and LuCaP136 + BPHF in all three in-vivo coinjection series. Therefore, the evidence for a stimulation of primary tumor growth in LuCaP136 xenografts by CAF-coinjection is very robust. A clear conclusion on the effect of fibroblast coinjection in LNCaP xenografts cannot be drawn yet as we only performed one coinjection experiment, which showed a stimulation of primary tumor growth by all fibroblasts compared to the injection of LNCaP cells alone but no differences in serum PSA values. Concerning metastatic spread we had more heterogeneous results. The number of lymph node metastases was significantly higher in LuCaP136 + CAF1 vs. LuCaP136 + NCAF1 (*p* < 0.03), in LuCaP136 + CAF2 vs. LuCaP136 + NCAF2/ LuCaP136 + BPHF2/ LuCaP136 alone (each *p* < 0.05) and in LuCaP136 + CAF3 vs. LuCaP136 + NCAF3 (*p* = 0.01). The frequency of lung metastases was significantly higher in LuCaP136 + CAF3 vs. LuCaP136 + NCAF3 and LuCaP136 alone (both *p* < 0.05). A non-significant trend towards a higher frequency of lung metastases was seen in LuCaP136 + CAF1 vs. LuCaP136 + NCAF1/ LuCaP136 alone (both *p* = 0.07). So in conclusion, a significant stimulation of metastatic spread to lymph nodes and lungs by CAF-coinjection in LuCaP136 xenografts was observed in some but not all comparisons. In LNCaP xenografts there were no statistically significant effects though the absolute numbers observed in the different groups look promising (for example lung metastases in 5/7 animals in the LNCaP + CAF1 group vs. 1/7 in the LNCaP alone group and both 2/7 in the LNCaP + NCAF1 and the LNCaP + BPHF1 group). To get more reliable results on the stimulation of metastatic spread to lymph nodes and lungs, the number of mice should be higher in future validation experiments.

An interesting observation was the differential effect of the diverse fibroblast populations on primary tumor growth and metastatic spread to regional lymph nodes and lungs in LuCaP136 and LNCaP xenografts. In LuCaP136 xenografts, CAFs stimulated primary tumor growth, while NCAF and BPHF coinjections had no effect compared to the injection of LuCaP136 cells alone. In contrast, in LNCaP xenografts, CAF, NCAF and BPHF coinjections all stimulated primary tumor growth in a similar manner compared to the injection of LNCaP cells alone with no difference between the different fibroblast subtypes. Concerning the influence of fibroblast coinjection on the development of metastases, we observed a stimulation of metastatic spread to regional lymph nodes and lungs by CAF coinjection compared to injection of tumor cells alone as well as the coinjection with the other two fibroblast types in both xenograft types though only the results in LuCaP136 xenografts were statistically significant (as described in detail above). The reasons for these different effects – especially the ones observed on primary tumor growth—in LuCaP136 and LNCaP xenografts remain elusive. In contrast to the characteristics of LuCaP136 spheroids, which are described above, LNCaP cells grow as conventional monolayer culture, are androgen-sensitive, express an androgen receptor with a point mutation in the ligand binding domain leading to a broad steroid binding specificity and show no engraftment when injected intratibially. Hence, due to its three-dimensional growth and its biological characteristics, LuCaP136 might be the more representative *in-vitro* model. For future studies it may be interesting to also use LNCaP cells as three-dimensional spheroids and to analyze if this makes a difference regarding local and metastatic progression or the effect of fibroblast coinjection. However, in this pilot study we decided to use both cell lines in the form in which they were originally established and are stably growing in vitro. Of note, while both cell lines express PSA but have a quite different PSA-density (with LuCaP136 spheroids secreting much less PSA), PSA-values correlated well with sonographic tumor volumes in both xenografts enabling longitudinal tracking of tumor burden even when sophisticated small animal imaging tools are not available (Suppl. Figure 2C).

While the differential effects of fibroblast coinjection seen in LuCaP136 and LNCaP xenografts are interesting observations deserving further investigation, this was not the major focus of our study. As we primarily aimed to establish a representative orthotopic in vivo model in which the biological effects of cancer-associated fibroblasts can be further investigated, we further focused on LuCaP136 xenografts in our validation experiments since we observed the strongest effects of CAF coinjection there in the first coinjection experiment. We cannot directly proof if the observed stimulation of metastatic spread in CAF-groups is a direct effect of CAF coinjection or more a consequence of increased primary tumor size. However, though both factors may play a role for the increased incidence of metastases, we believe the first point to be biologically more important. While in some tumors like renal cell carcinoma the incidence of metastases is clearly correlated with primary tumor size^49^ this is not the case for prostate cancer. In contrast, it has been shown in several studies that CAFs are able to stimulate metastasis-associated biological features in nearby PCa cells like invasion, epithelial-to-mesenchymal transition (EMT), angiogenesis and stemness traits^33–36,50,51^.

When we examined the retrieved primary tumors by H&E histology as well as immunohistochemistry after ten weeks of follow-up, we could no longer observe human fibroblasts. This observation is consistent with the findings from PDX models, in which the human stromal cells are gradually and finally completely replaced by murine equivalents^52,53^. In addition, these results indicate that the fibroblast-induced effects observed in our study might occur early in the disease course, when the coinjected human fibroblasts are still present, which explains the early separation of the growth curves observed in LuCaP136 xenografts. To see stronger effects of CAFs and observe their effect over a longer time frame, one might either have to increase the fibroblast : cancer cell ratio as Mishra et al. did in their study (3:1)^38^ or use immortalized fibroblasts, which may show longer in-vivo survival compared to patient-derived primary cultures but are on the other hand less biologically representative. Additionally, to further elucidate the dynamic process of microenvironment remodeling (replacement of human fibroblasts by murine equivalents), it could be considered to sacrifice animals at different time points after coinjection and examine the retrieved tumors by immunohistochemistry or FACS analyses.

In our study, we detected an increased rate of lymph node and lung metastases after intraprostatic coinjection of CAFs, an interesting observation that deserves further investigation. While robust experimental evidence has shown the stimulatory effect of CAFs on primary tumor growth, little is known about their role in local progression and metastatic spread of PCa^54^. They might contribute to these processes, for example, by the secretion of matrix metalloproteinases or extracellular vesicles^55–57^. However, the molecular mechanisms involved in these complex interactions are still unclear to date.

In our study, we used small animal imaging (high-resolution 3D ultrasonography, in-vivo micro-CT, 9.4 T MRI) and histological/immunohistochemical evaluation of the removed organs to monitor metastatic progression. While these sophisticated tools allow for a sensitive and specific detection of most metastases, other techniques like bioluminescence or PCR for human-specific Alu-sequences could further improve the detection and exact quantification of metastatic disease in such a model.

Finally, the term “cancer-associated fibroblasts” probably refers to a fairly heterogeneous group of CAF subtypes that remain to be clearly characterized molecularly and functionally to improve our understanding of their role in TME – cancer cell interactions^58–60^. While some groups tried to classify CAFs into distinct subgroups (e.g. CAF 1, 2 and 3), we believe such a categorization to be somewhat arbitrary as the evolution of CAFs during the progression of PCa is probably a continuous, spatially and temporally heterogeneous process. Accordingly, our primary cultures can be expected to be a mixture of different CAF subtypes and we did not further characterize them by staining additional markers (e.g. FAP, CD49b, FSP1) since these results would have no influence on the key messages of our study. Nonetheless, a deeper molecular characterization of a panel of fibroblast primary cultures including the ones used in this study has been previously done on the gene expression level showing dysregulated expression of various genes in CAFs compared to NCAFs and BPHFs^61^. For example, the genes *SDF1*, *TGFβ*, *PDGFRβ* and *FAP* were significantly upregulated in CAFs (Suppl. Figure 8)^61^. Apart from that, after having established a valuable in vivo model to examine the effects of CAFs on PCa progression in this study, it may be worthwhile to focus in detail on the differential role of putative CAF subtypes in future investigations.

In conclusion, we for the first time successfully established a versatile in vivo model that representatively displays the stimulatory effects of cancer-associated fibroblasts on prostate cancer progression by combining three-dimensional spheroids, patient-derived fibroblast primary cultures and orthotopic xenografts. This model provides a valuable tool to further unravel the molecular mechanisms involved in the crosstalk between cancer cells and their microenvironment, which plays an important role in prostate cancer progression, metastatic spread and therapeutic response.

## Supplementary information

Supplementary file 1.
