# Supplementary material for: Cancer-associated fibroblasts stimulate primary tumor growth and metastatic spread in an orthotopic prostate cancer xenograft model
Source: Sci Rep. 2020 Jul 28;10:12575. doi: 10.1038/s41598-020-69424-x (PMC7387494; doi:10.1038/s41598-020-69424-x)
Supplement: Supplementary file 1 [file 41598_2020_69424_MOESM1_ESM.pptx]

## Slide 1
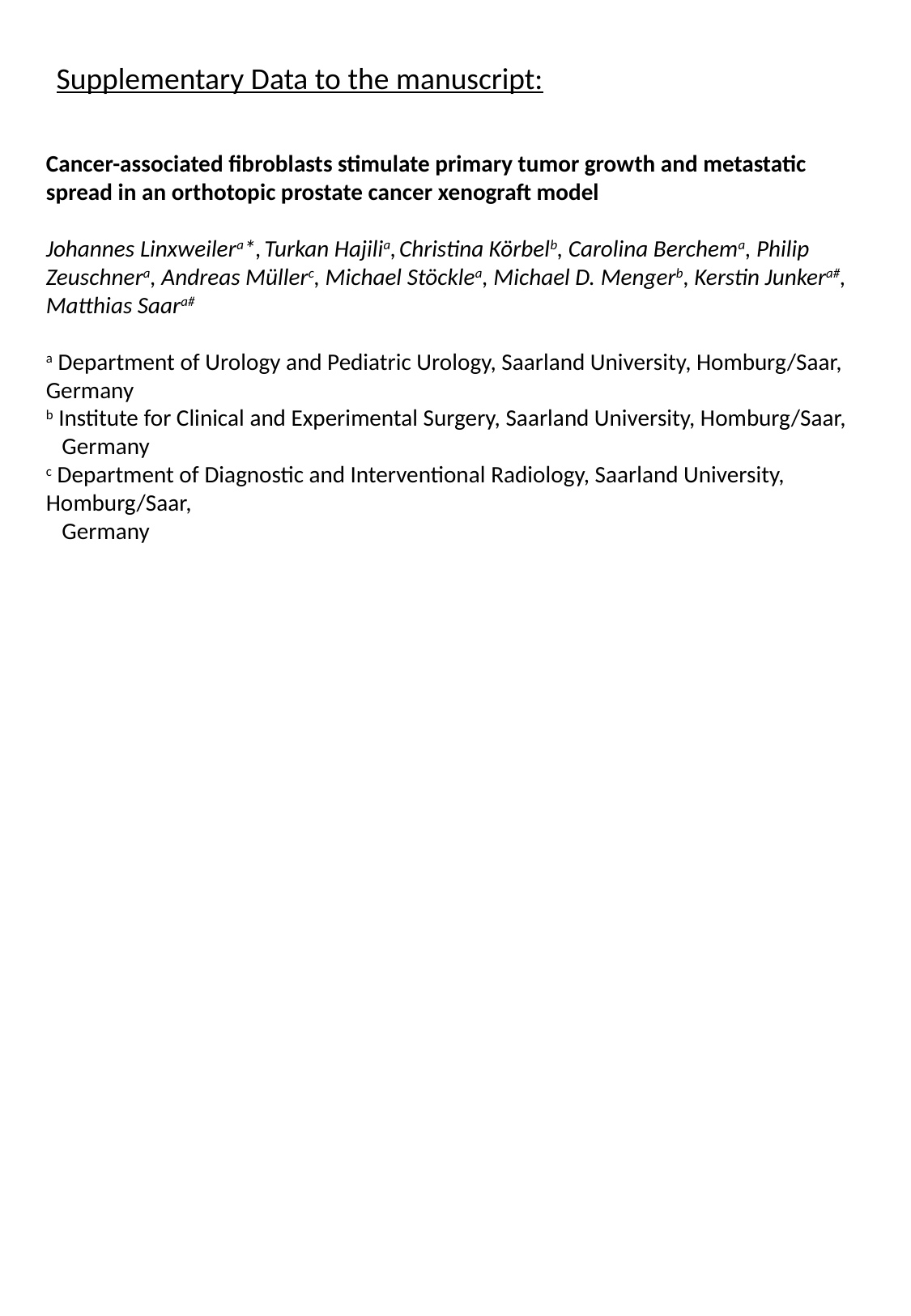

Supplementary Data to the manuscript:
Cancer-associated fibroblasts stimulate primary tumor growth and metastatic spread in an orthotopic prostate cancer xenograft model
Johannes Linxweilera*, Turkan Hajilia, Christina Körbelb, Carolina Berchema, Philip Zeuschnera, Andreas Müllerc, Michael Stöcklea, Michael D. Mengerb, Kerstin Junkera#, Matthias Saara#
a Department of Urology and Pediatric Urology, Saarland University, Homburg/Saar, Germany
b Institute for Clinical and Experimental Surgery, Saarland University, Homburg/Saar,
 Germany
c Department of Diagnostic and Interventional Radiology, Saarland University, Homburg/Saar,
 Germany

## Slide 2
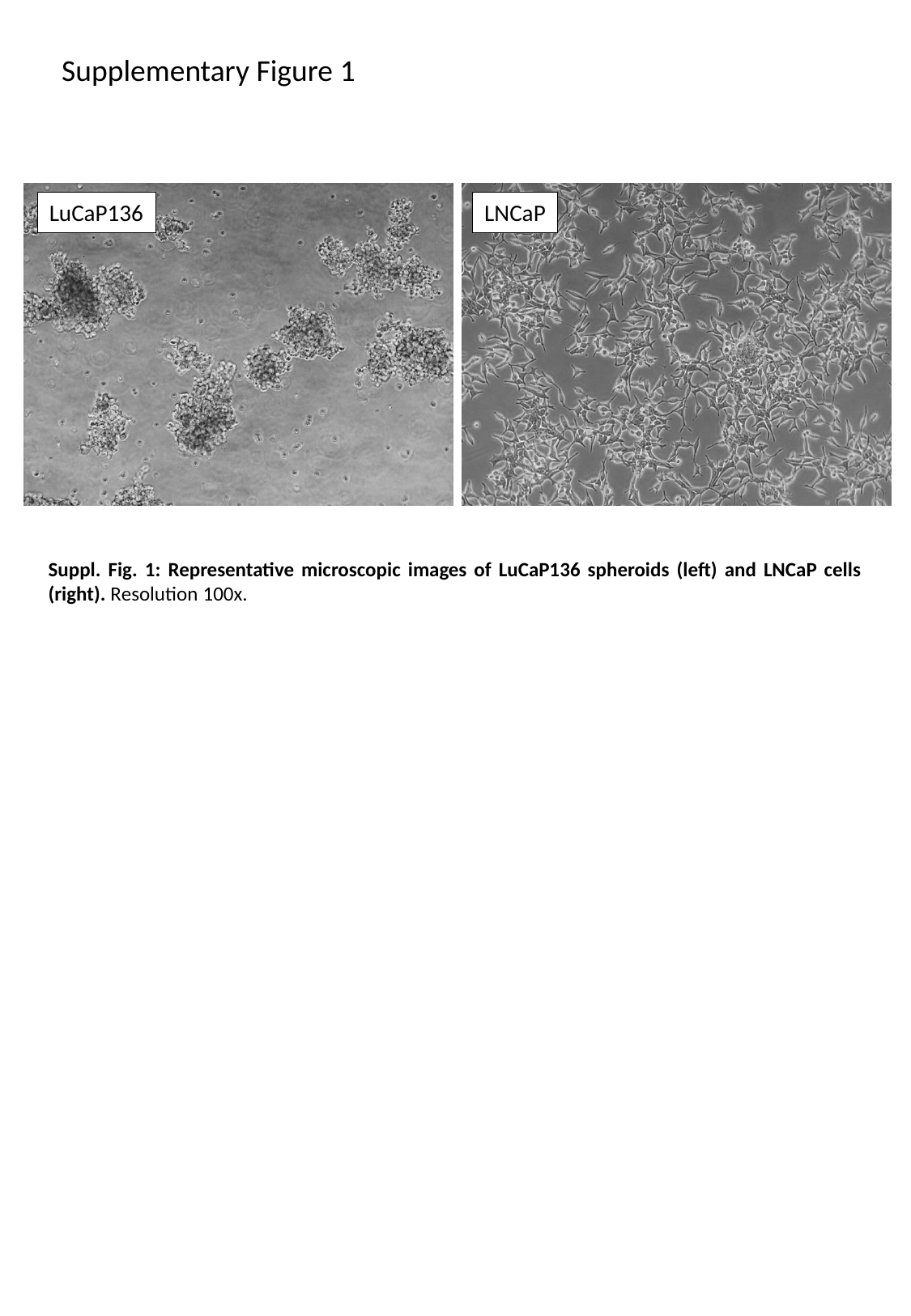

Supplementary Figure 1
LNCaP
LuCaP136
Suppl. Fig. 1: Representative microscopic images of LuCaP136 spheroids (left) and LNCaP cells (right). Resolution 100x.

## Slide 3
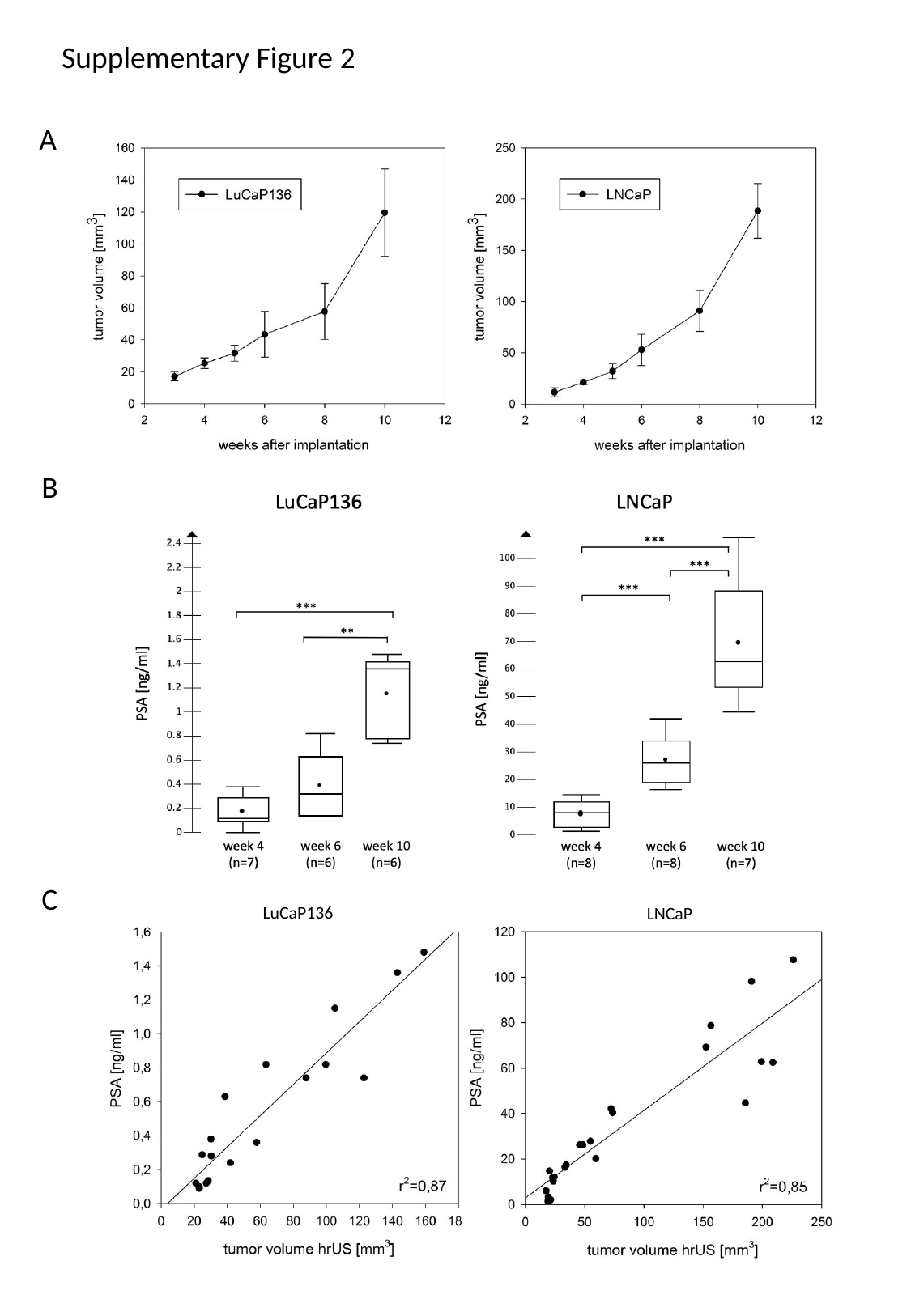

Supplementary Figure 2
A
B
C
LuCaP136
LNCaP

## Slide 4
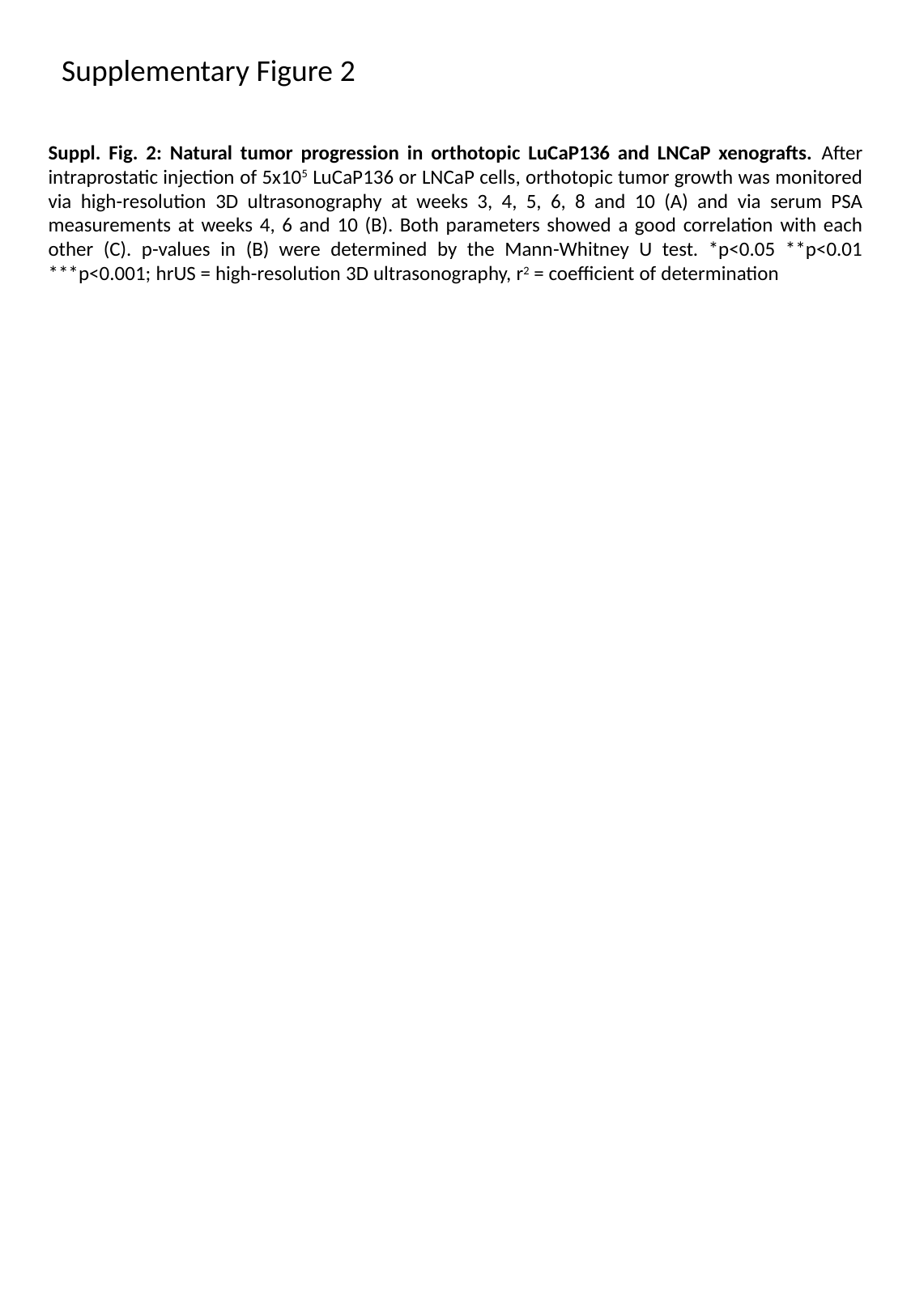

Supplementary Figure 2
Suppl. Fig. 2: Natural tumor progression in orthotopic LuCaP136 and LNCaP xenografts. After intraprostatic injection of 5x10^5^ LuCaP136 or LNCaP cells, orthotopic tumor growth was monitored via high-resolution 3D ultrasonography at weeks 3, 4, 5, 6, 8 and 10 (A) and via serum PSA measurements at weeks 4, 6 and 10 (B). Both parameters showed a good correlation with each other (C). p-values in (B) were determined by the Mann-Whitney U test. *p<0.05 **p<0.01 ***p<0.001; hrUS = high-resolution 3D ultrasonography, r2 = coefficient of determination

## Slide 5
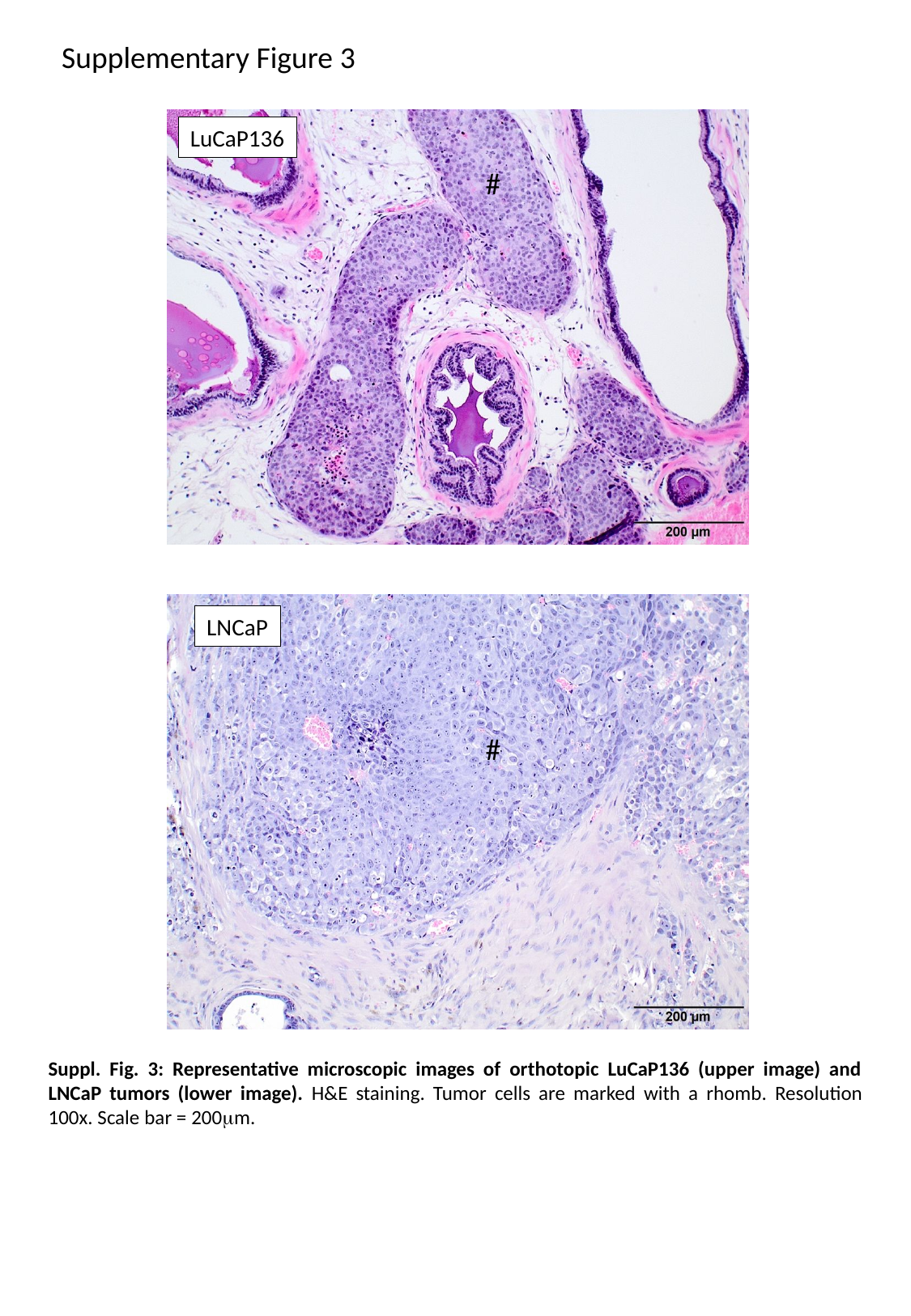

Supplementary Figure 3
LuCaP136
#
LNCaP
#
Suppl. Fig. 3: Representative microscopic images of orthotopic LuCaP136 (upper image) and LNCaP tumors (lower image). H&E staining. Tumor cells are marked with a rhomb. Resolution 100x. Scale bar = 200mm.

## Slide 6
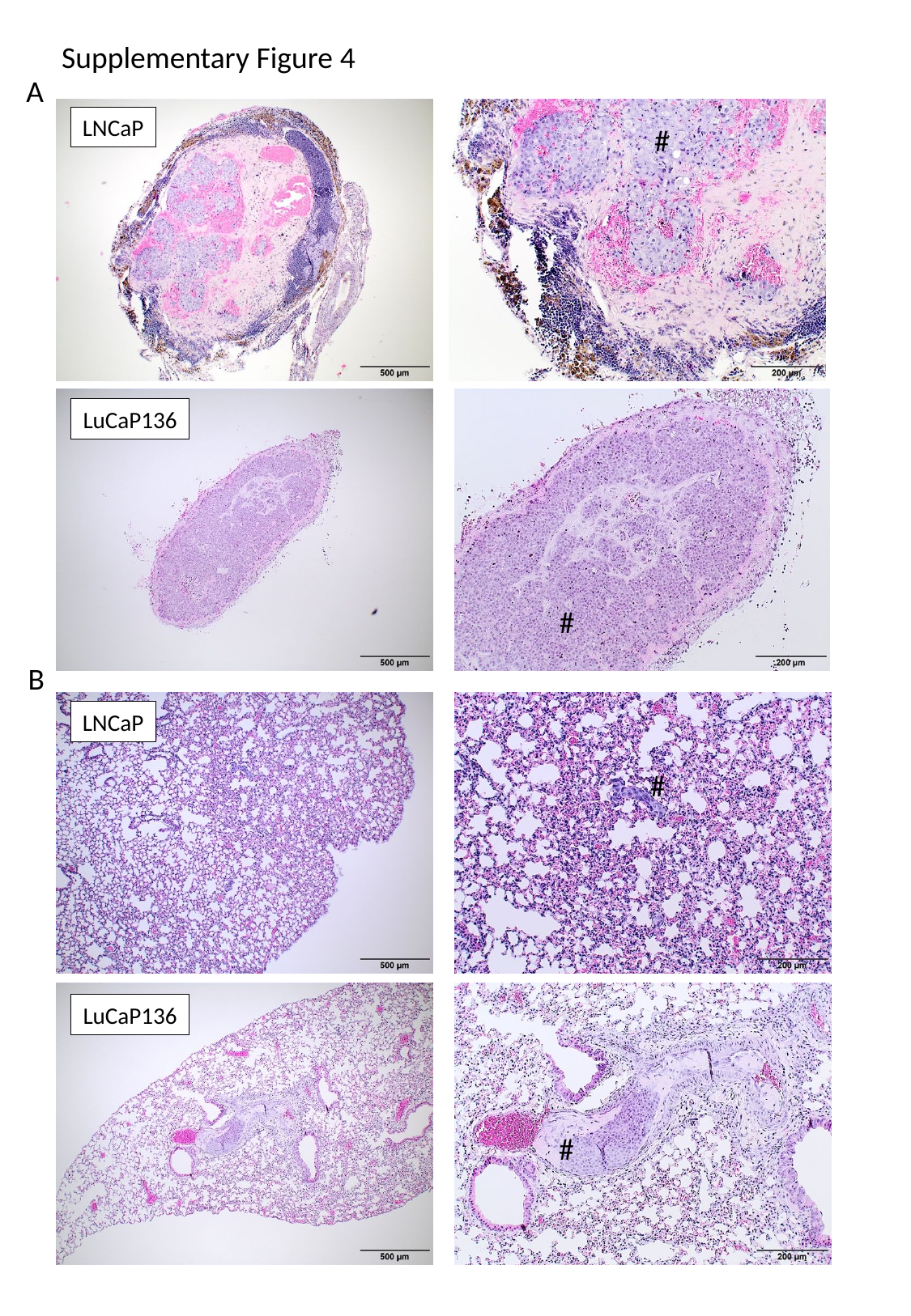

Supplementary Figure 4
A
LNCaP
#
LuCaP136
#
B
LNCaP
#
LuCaP136
#

## Slide 7
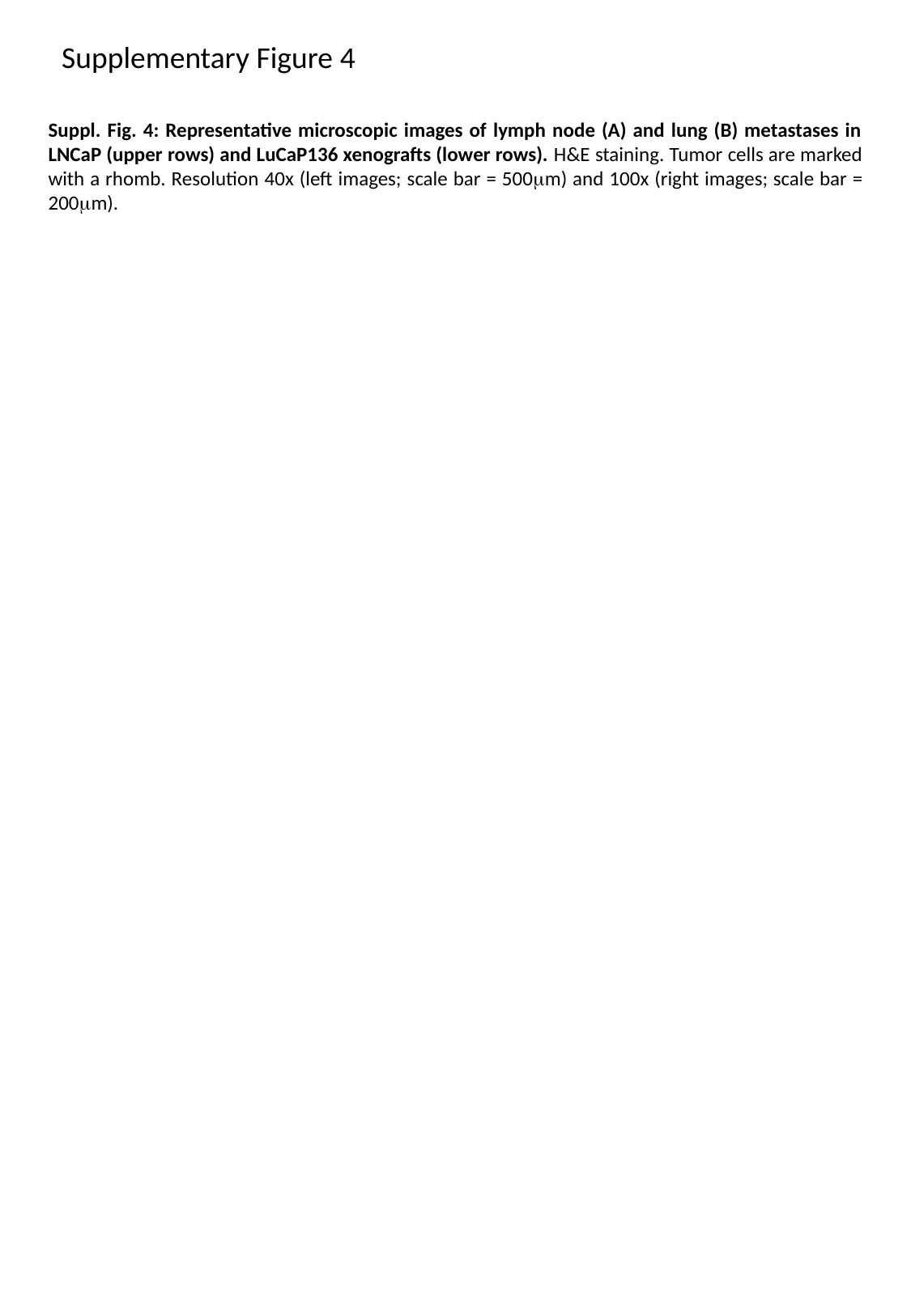

Supplementary Figure 4
Suppl. Fig. 4: Representative microscopic images of lymph node (A) and lung (B) metastases in LNCaP (upper rows) and LuCaP136 xenografts (lower rows). H&E staining. Tumor cells are marked with a rhomb. Resolution 40x (left images; scale bar = 500mm) and 100x (right images; scale bar = 200mm).

## Slide 8
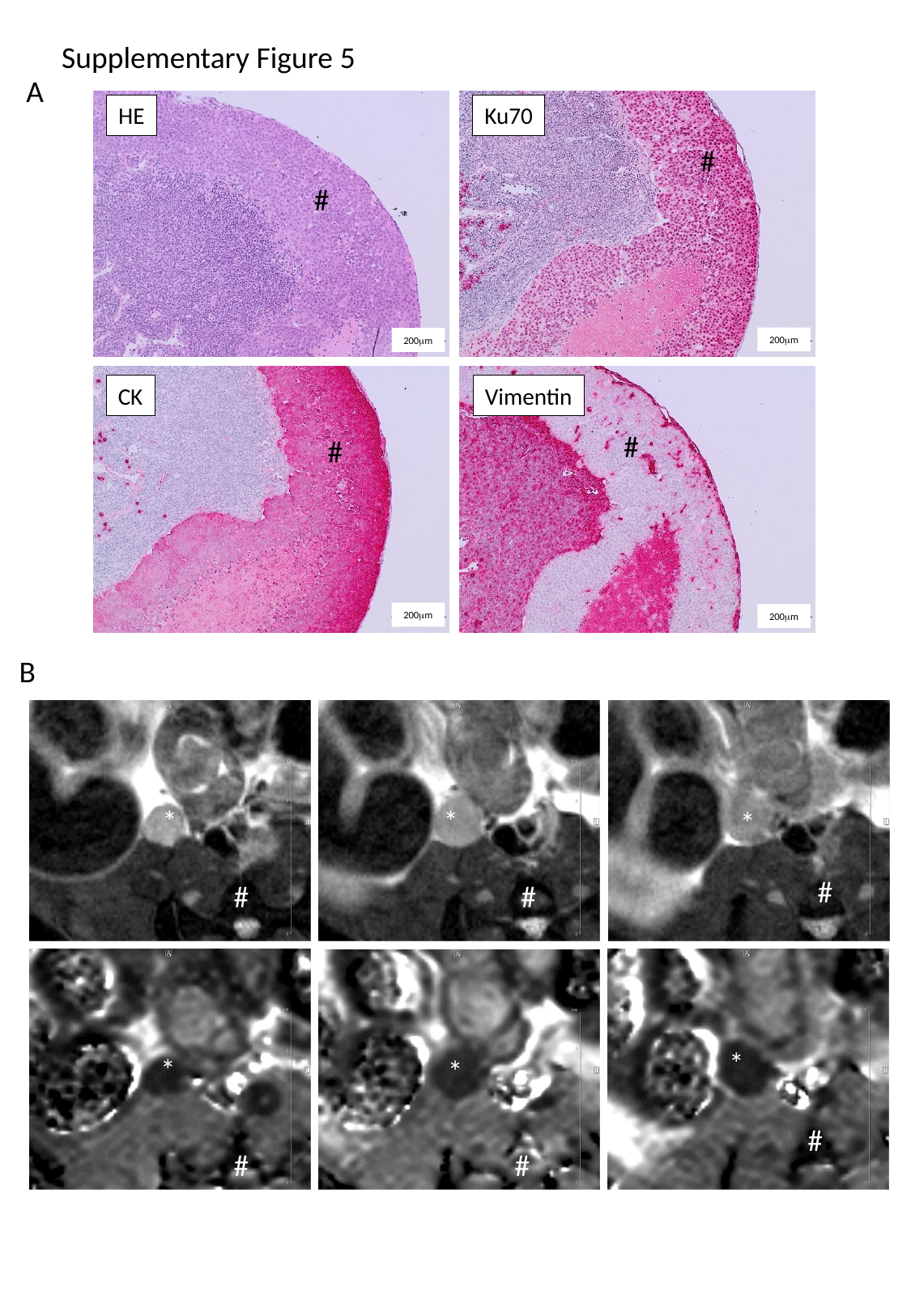

Supplementary Figure 5
A
HE
Ku70
#
#
200mm
200mm
CK
Vimentin
#
#
200mm
200mm
B
*
*
*
#
#
#
*
*
*
#
#
#

## Slide 9
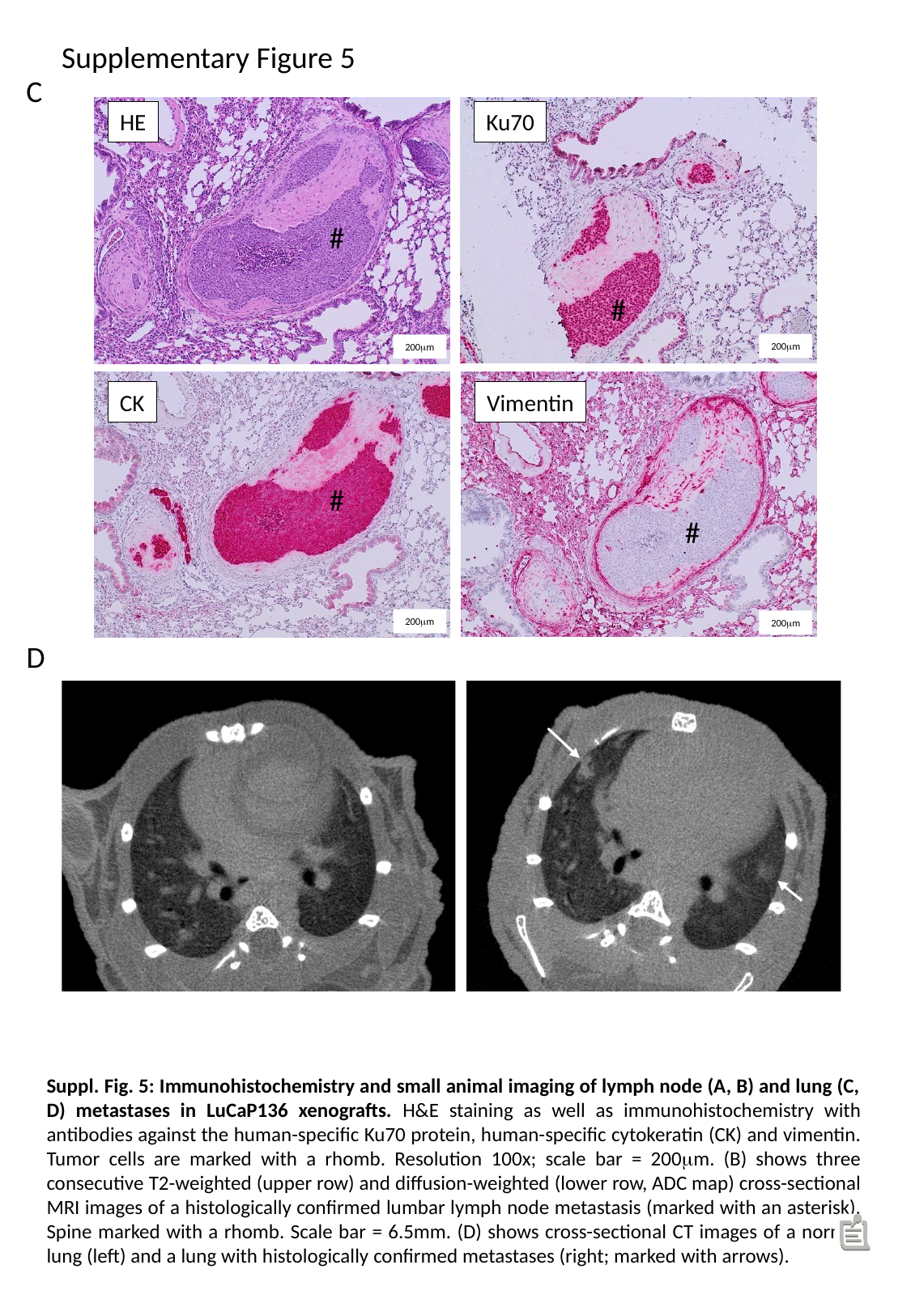

Supplementary Figure 5
C
HE
Ku70
#
#
200mm
200mm
CK
Vimentin
#
#
200mm
200mm
D
Suppl. Fig. 5: Immunohistochemistry and small animal imaging of lymph node (A, B) and lung (C, D) metastases in LuCaP136 xenografts. H&E staining as well as immunohistochemistry with antibodies against the human-specific Ku70 protein, human-specific cytokeratin (CK) and vimentin. Tumor cells are marked with a rhomb. Resolution 100x; scale bar = 200mm. (B) shows three consecutive T2-weighted (upper row) and diffusion-weighted (lower row, ADC map) cross-sectional MRI images of a histologically confirmed lumbar lymph node metastasis (marked with an asterisk). Spine marked with a rhomb. Scale bar = 6.5mm. (D) shows cross-sectional CT images of a normal lung (left) and a lung with histologically confirmed metastases (right; marked with arrows).

## Slide 10
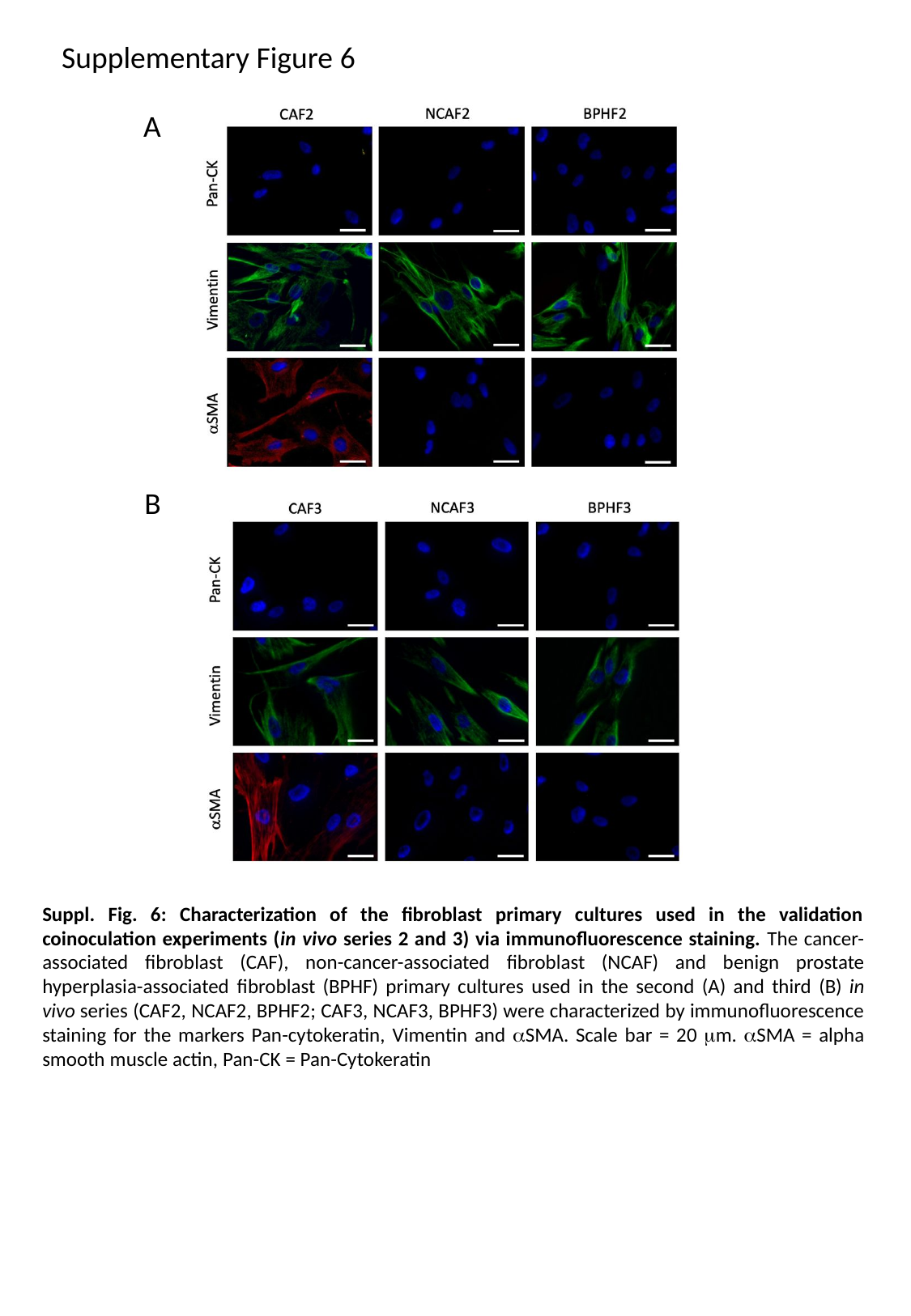

Supplementary Figure 6
A
B
Suppl. Fig. 6: Characterization of the fibroblast primary cultures used in the validation coinoculation experiments (in vivo series 2 and 3) via immunofluorescence staining. The cancer-associated fibroblast (CAF), non-cancer-associated fibroblast (NCAF) and benign prostate hyperplasia-associated fibroblast (BPHF) primary cultures used in the second (A) and third (B) in vivo series (CAF2, NCAF2, BPHF2; CAF3, NCAF3, BPHF3) were characterized by immunofluorescence staining for the markers Pan-cytokeratin, Vimentin and αSMA. Scale bar = 20 μm. αSMA = alpha smooth muscle actin, Pan-CK = Pan-Cytokeratin

## Slide 11
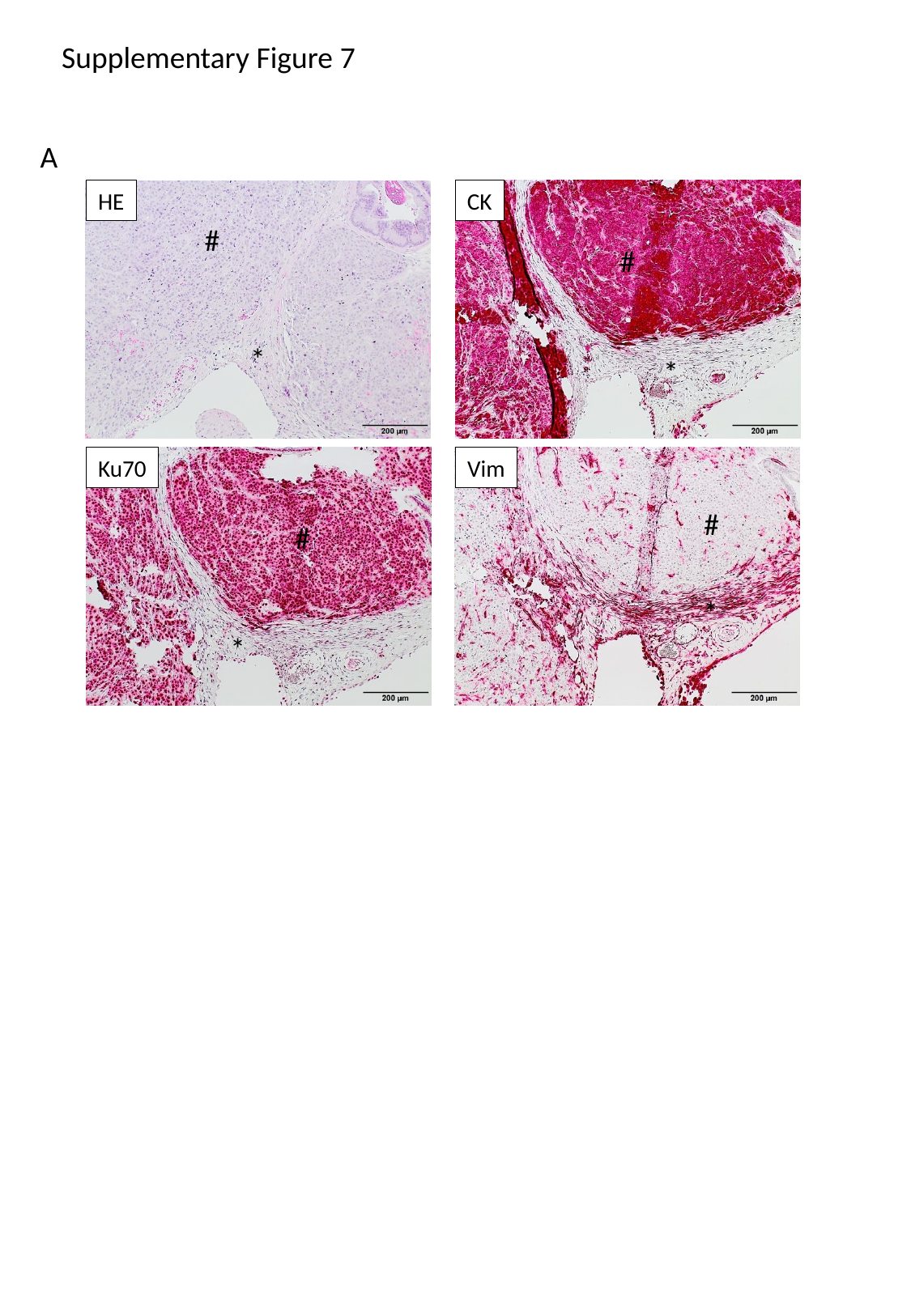

Supplementary Figure 7
A
HE
CK
#
#
*
*
Ku70
Vim
#
#
*
*
*

## Slide 12
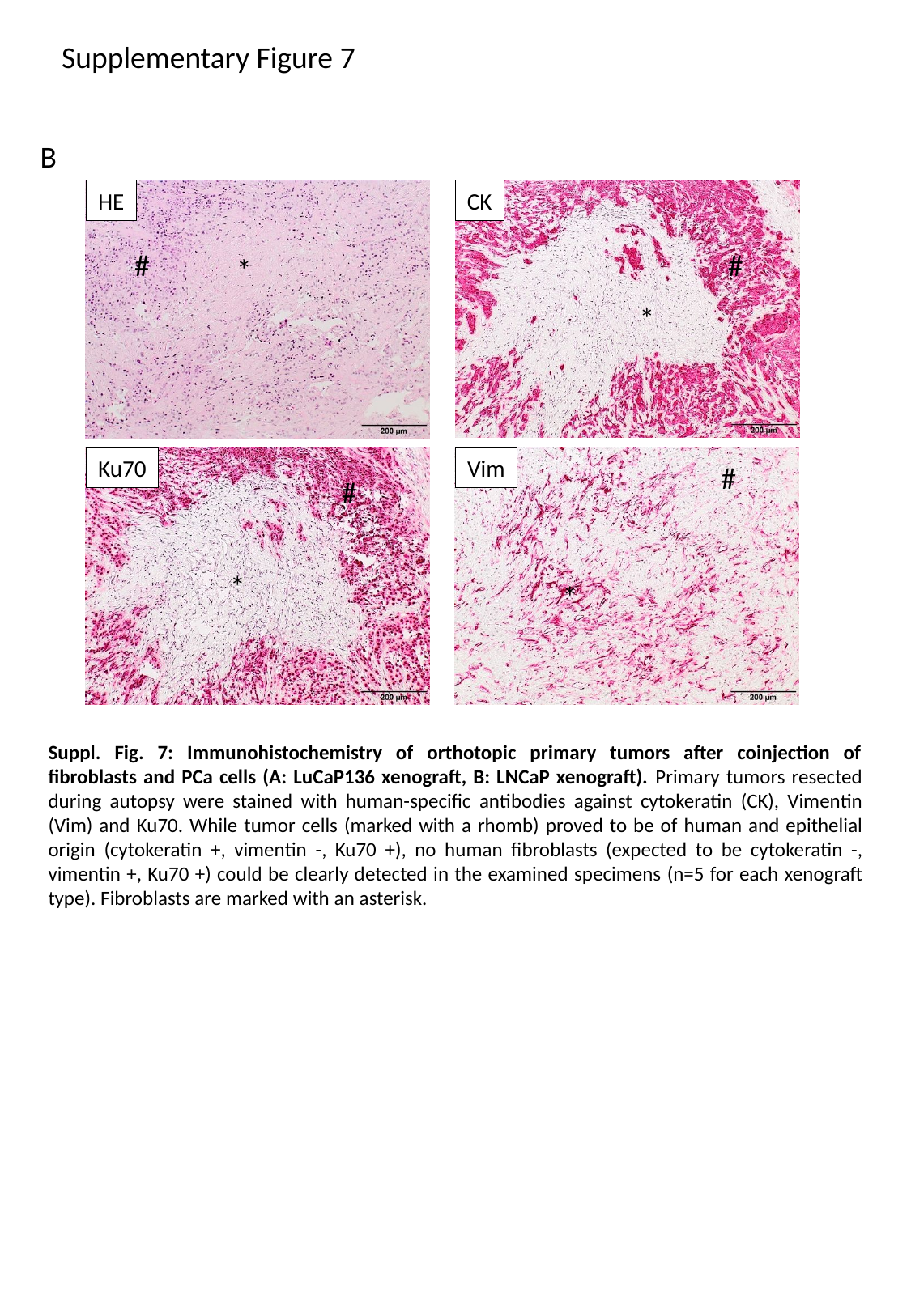

Supplementary Figure 7
B
HE
CK
#
#
*
*
Ku70
Vim
#
#
*
*
Suppl. Fig. 7: Immunohistochemistry of orthotopic primary tumors after coinjection of fibroblasts and PCa cells (A: LuCaP136 xenograft, B: LNCaP xenograft). Primary tumors resected during autopsy were stained with human-specific antibodies against cytokeratin (CK), Vimentin (Vim) and Ku70. While tumor cells (marked with a rhomb) proved to be of human and epithelial origin (cytokeratin +, vimentin -, Ku70 +), no human fibroblasts (expected to be cytokeratin -, vimentin +, Ku70 +) could be clearly detected in the examined specimens (n=5 for each xenograft type). Fibroblasts are marked with an asterisk.

## Slide 13
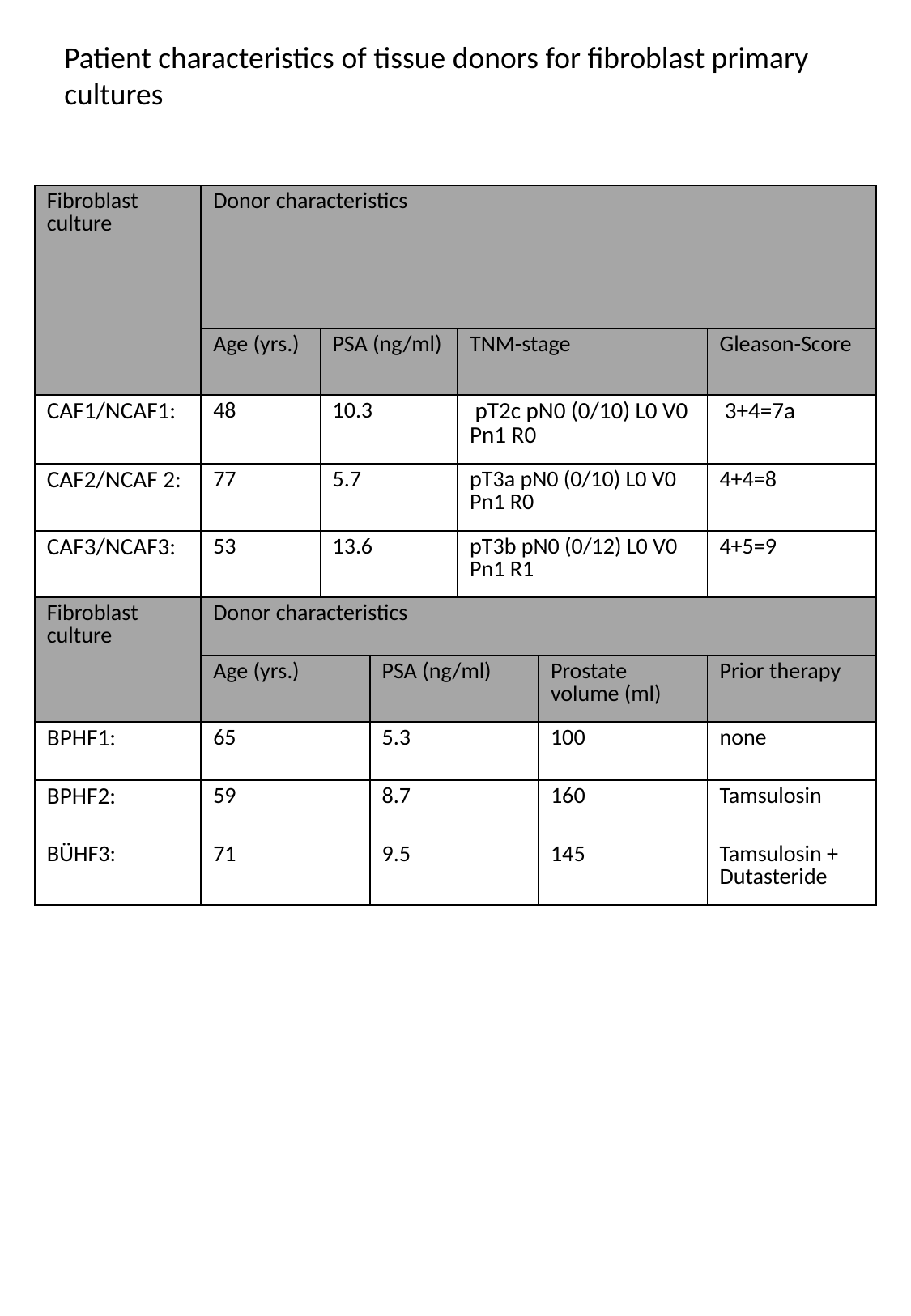

Patient characteristics of tissue donors for fibroblast primary
cultures
| Fibroblast culture | Donor characteristics | | | | | |
| --- | --- | --- | --- | --- | --- | --- |
| | Age (yrs.) | PSA (ng/ml) | PSA (ng/ml) | TNM-stage | TNM-stage | Gleason-Score |
| CAF1/NCAF1: | 48 | 10.3 | 10.3 | pT2c pN0 (0/10) L0 V0 Pn1 R0 | pT2c pN0(0/10) L0 V0 Pn1 R0 | 3+4=7a |
| CAF2/NCAF 2: | 77 | 5.7 | 5.7 | pT3a pN0 (0/10) L0 V0 Pn1 R0 | pT3a pN0 (0/10) L0 V0 Pn1 R0 | 4+4=8 |
| CAF3/NCAF3: | 53 | 13.6 | | pT3b pN0 (0/12) L0 V0 Pn1 R1 | | 4+5=9 |
| Fibroblast culture | Donor characteristics | | | | | |
| | Age (yrs.) | | PSA (ng/ml) | | Prostate volume (ml) | Prior therapy |
| BPHF1: | 65 | | 5.3 | | 100 | none |
| BPHF2: | 59 | | 8.7 | | 160 | Tamsulosin |
| BÜHF3: | 71 | | 9.5 | | 145 | Tamsulosin + Dutasteride |

## Slide 14
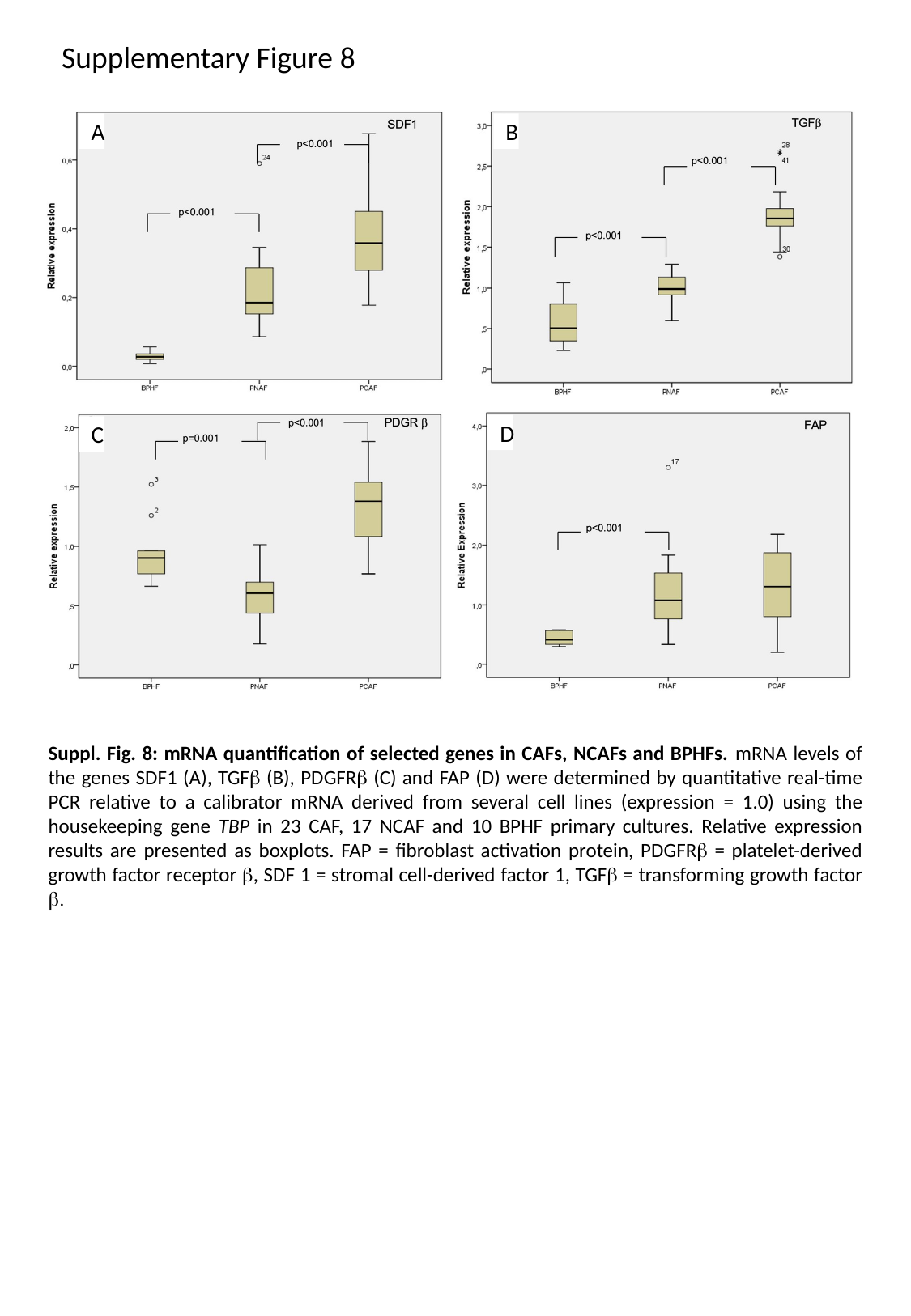

Supplementary Figure 8
A
B
D
C
Suppl. Fig. 8: mRNA quantification of selected genes in CAFs, NCAFs and BPHFs. mRNA levels of the genes SDF1 (A), TGFb (B), PDGFRb (C) and FAP (D) were determined by quantitative real-time PCR relative to a calibrator mRNA derived from several cell lines (expression = 1.0) using the housekeeping gene TBP in 23 CAF, 17 NCAF and 10 BPHF primary cultures. Relative expression results are presented as boxplots. FAP = fibroblast activation protein, PDGFRb = platelet-derived growth factor receptor b, SDF 1 = stromal cell-derived factor 1, TGFb = transforming growth factor b.
